# Synthesis and Structures of Carbon Nanohoops Containing Three Picene Units with Each Having Two Carbomethoxy Substituents

**DOI:** 10.1021/acs.orglett.5c01384

**Published:** 2025-06-17

**Authors:** Liu Li, Stephen M. Long, Nathaniel J. Selvaraj, Brian S. Dolinar, Brian V. Popp, Kung K. Wang

**Affiliations:** C. Eugene Bennett Department of Chemistry, 5631 West Virginia University, Morgantown, West Virginia 26506, United States

## Abstract

Carbon nanohoops are an emerging class of cyclic conjugated molecules that have drawn significant attention for their unique optoelectronic properties and potential applications in nanomaterials. We report herein the synthesis of two novel carbon nanohoops with structures as [9]­cycloparaphenylene ([9]­CPP) derivatives having three picene units with each substituted by two carbomethoxy groups. A substrate bearing a hexahydropicene unit with the two bromophenyl groups *cis* to each other through the Diels–Alder reaction with dimethyl acetylenedicarboxylae was prepared in four steps from 6-bromo-1-tetralone. A subsequent Ni­(cod)_2_/bpy-mediated macrocyclization reaction followed by a DDQ-promoted oxidation step of the central six-membered ring and the dimethylene linkages produced a pair of cyclic *anti*- and *syn*-trimers because of the slow rotation of the dicarbomethoxy-substituted picene units. This work not only introduces new CPP architecture but also provides insights into the potential extension of this methodology toward the synthesis of cyclophenacenes as carbon nanobelts.

## Graphical abstract

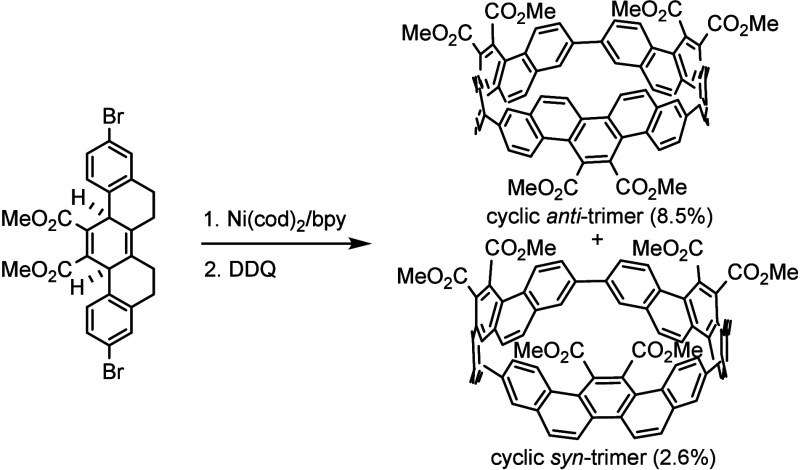

Carbon nanohoops are cyclic π-conjugated molecules composed solely of benzene units linked through *para* positions.^1^ These structures are of immense interest as they represent the smallest structural units of armchair carbon nanotubes (CNTs) and serve as ideal models for investigating curvature-induced strain,^2^ aromaticity,^3^ size-dependent optoelectronic properties,^4^ and host–guest interactions.^5^ Since the first successful synthesis of carbon nanohoops in 2008,^1a^ research has expanded to incorporate various functional groups, including heterocycles^6^ and extended aromatic systems,^7–9^ into the carbon nanohoop framework. These modifications have opened avenues for tailoring their electronic, photophysical, and host–guest properties, making carbon nanohoops valuable in fields ranging from molecular electronics to supramolecular chemistry.^10^

The carbon nanohoop derivatives containing fused polycyclic aromatic hydrocarbons (PAHs), such as pyrene,^7^ naphthalene,^8^ and other systems,^9^ have garnered significant attention due to their enhanced rigidity, unique photophysical properties, and potential applications in organic electronics. Despite these advancements, the incorporation of picene, a PAH containing five fused benzene rings known for its semiconducting properties and stability,^11^ into the carbon nanohoop structure has remained unexplored.

We describe herein the synthesis of two carbon nanohoops with structures as [9]­CPP derivatives having three picene units. The synthesis was initiated from 6-bromo-1-tetralone (**1**) using a method reported previously (Scheme 1).^12^ The enolate of **1,** generated by treatment with LDA at −78 °C in THF, underwent a ferric chloride-promoted oxidative coupling reaction to afford diketone **2** in 80% isolated yield. Because of the presence of two chiral centers at the alpha positions of diketone **2**, two diastereomers, *RR*/*SS* and *RS*/*SR* pairs, were produced as indicated by the ^1^H and ^13^C NMR spectra. Subsequent reduction of **2** with NaBH_4_ in methanol produced diol **3**, which was used directly in the following step, in part because it contains various isomers. The crude diol **3** was treated with PBr_3_ in pyridine at 70 °C, resulting in the formation of diene **4** in 55% isolated yield over two steps from **2**. Conducting the dehydration step under a lower temperature (0 °C to rt) gave a diminished yield of **4**. The Diels–Alder reaction between diene **4** and dimethyl acetylenedicarboxylate (**5**) was performed in toluene at 90 °C for 16 h, providing the cycloaddition adduct **6** as a hexahydropicene derivative in 59% isolated yield. Elevated reaction temperatures led to significant alkene shift, forming undesirable byproducts. While the Diels–Alder adduct **6** was very stable in air, it was sensitive to bases and forms the aromatized central ring as can be expected. The Diels–Alder rection also places the two bromophenyl groups *cis* to each other in the central 6-membered diene system, which is important for the following macrocyclization reaction.^13^

**Scheme 1:**
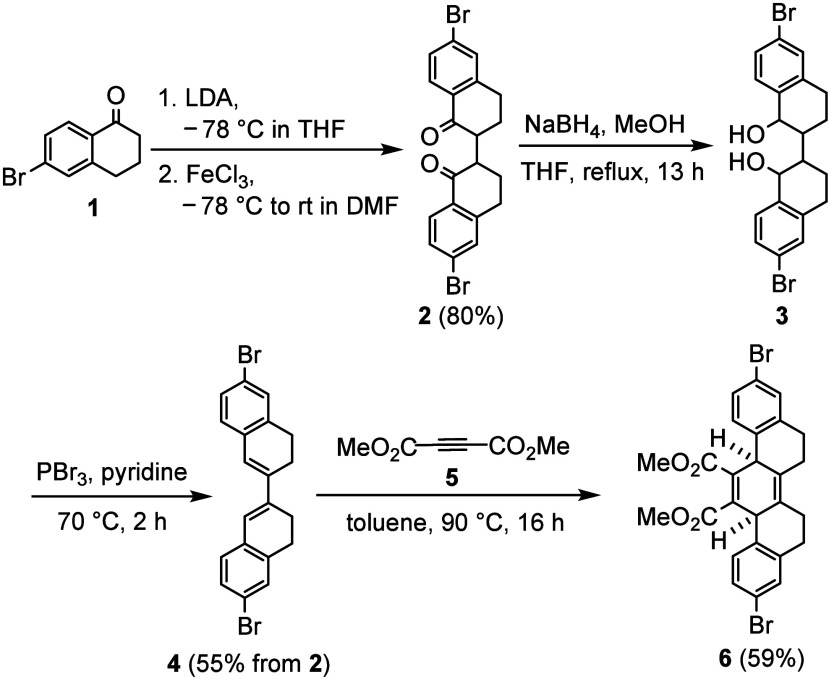
Synthesis of Hexahydropicene **6**

The molecular structure of **6** was confirmed by the X-ray crystallography (Figure 1), and the X-ray structure of **6** reveals that the included angle of the two bonds attaching the two bromophenyl groups to the central diene ring is 54.2° in the crystal lattice,^14^ which is much smaller than the cases reported previously.^15^ The presence of the two additional dimethylene linkages may be responsible for the decreased included angle, holding the two bromophenyl groups in place without free rotation.

**Figure 1:**
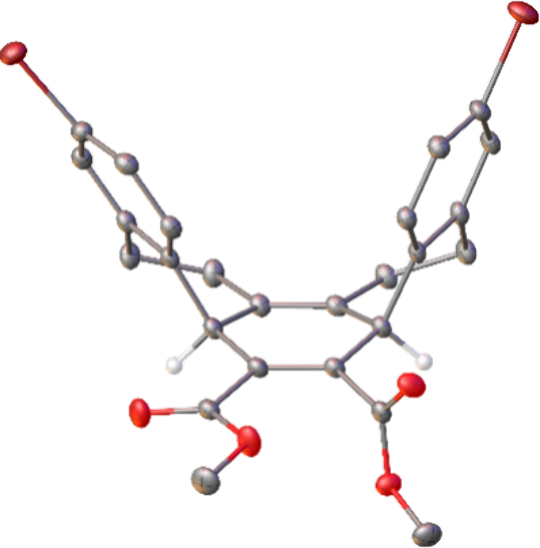
X-ray crystal structure of hexahydropicene **6**.

With hexahydropicene **6** in hand, the macrocyclization reaction was conducted with 1.5 equiv of Ni­(cod)_2_/bpy (cod: 1,5-cyclooctadiene; bpy: 2,2′-bipyridyl) in THF (10 mM of **6**) (Scheme 2). The use of 1.5 equiv of Ni­(cod)_2_/bpy for macrocyclization was reported to produce the best yield for the CPP formation.^15^ Upon completion of the Ni­(cod)_2_/bpy reaction, the reaction products were not purified, but were used directly in the subsequent oxidation step with 2,3-dichloro-5,6-dicyano-1,4-benzoquinone (DDQ) in chlorobenzene at 120 °C to provide a pair of isomers of cyclic *anti*-trimer **7** (8.5% isolated yield) and *syn*-trimer **7** (2.6% isolated yield) containing three picene units with each having two carbomethoxy substituents.

**Scheme 2:**
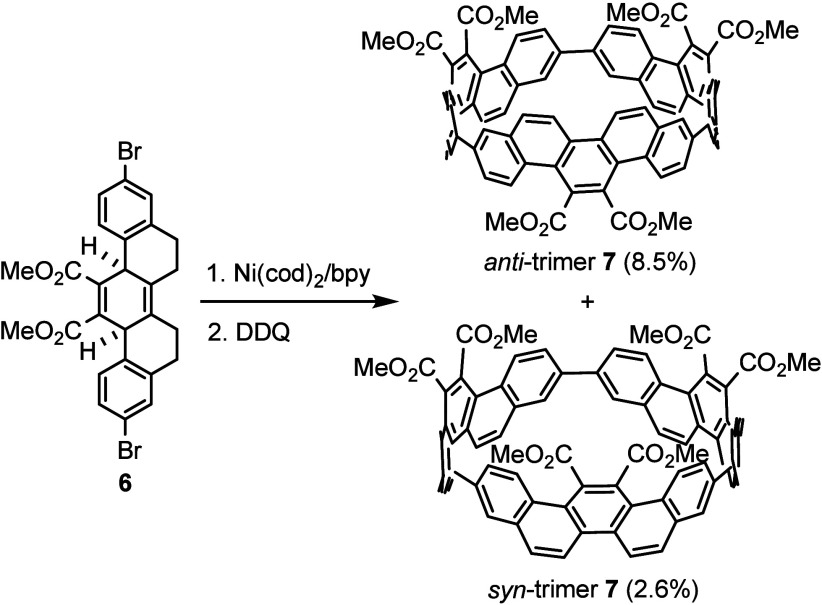
Macrocyclization and Subsequent Oxidation of **6** To Form a Pair of Cyclic Isomers of *anti*-Trimer **7** and *syn*-Trimer **7** Containing Three Picene Units

This result differs from the previous reported synthesis of a [9]­CPP derivative in which only a single isomer was obtained.^16^ In the previous case having only benzene rings without the presence of ethylene linkages as in the picene unit, the rate of rotation of the benzene groups having the two carbomethoxy groups is faster than the NMR time scale. As a result, only one cyclic isomeric structure was observed by the NMR spectra. The presence of three picene units in *anti*-trimer **7** and *syn*-trimer **7** greatly slows the rotation rate, allowing the NMR spectra to show two distinct cyclic isomers. The ^1^H NMR spectrum of *anti*-trimer **7** showed no interconversion between *anti*-trimer **7** and *syn*-trimer **7** at room temperature for at least 2 weeks.

Although the dimethylene linkages in **6** likely increase the ring strain during macrocyclization, the smaller included angle of the two bonds attaching the two bromophenyl groups to the central diene ring may facilitate the macrocyclization process. No dimers or other oligomers of larger rings were found. The DDQ-promoted benzylic oxidation of the dimethylene linkages to form the three picene units was similar to what was observed previously.^16^

The two cyclic trimers were initially characterized by high-resolution mass spectroscopy (Supporting Information, Figure S2). The high-resolution mass spectra of both compounds showed clearly that their isotopic peak patterns matched the calculated ones. Further structural confirmation for *anti*-trimer **7** was achieved using ^1^H NMR spectra. The molecular structure of *anti*-trimer **7** has a symmetry plane. In CDCl_3_, the integration of peaks at ca. δ 3.96 ppm, corresponding to the methoxy groups, indicated an approximately 2:1 ratio with two of the three methoxy groups overlapping with each other. However, as the methoxy peaks were not well separated in CDCl_3_, further analysis in DMSO-*d*_6_ and C_6_D_6_ revealed three sharp, distinct methoxy singlets between δ 3.89–3.93 and 3.61–3.66 ppm, respectively, with equal integration ratio of 1:1:1 as can be expected. In the aromatic region in C_6_D_6_, the peaks were reasonably well separated despite the low solubility of *anti*-trimer **7** in C_6_D_6_. Three distinct doublets (2 H each) for protons in the aromatic bay region were observed between 8.34–8.60 ppm. Additional characteristic signals included a singlet (2 H, *anti* picene unit) at δ 7.89 ppm, overlapping with the left-hand side of a doublet, and overlapping singlets (4 H) at δ 7.74 ppm. In addition, an overlapping doublet (2 H) at δ 7.88 ppm, overlapping doublets (4 H) as a triplet at δ 7.82 and 7.80 ppm, and a well-separated doublet (2 H) at δ 7.416 ppm were observed. The remaining five doublets (10 H) in the aromatic region between δ 7.56–7.48 ppm were also observed, accounting for a total of 30 ^1^H NMR signals in the aromatic region. The ^13^C NMR spectrum in DMSO-*d*_6_ clearly showed the presence of three different carbonyl carbons of the carbomethoxy groups. The rest of the ^13^C NMR spectrum was consistent with the presence of a symmetry plan.

For *syn*-trimer **7**, the molecular structure was also confirmed by the ^1^H NMR spectrum in DMSO-*d*_6_. Because of the presence of a *C*_*3*_ axis, the resulting molecular structure had of a higher symmetry element and only one methoxy singlet appearing at δ 3.90 ppm was observed. In the aromatic region, the ^1^H NMR spectrum also showed much simpler signals with a singlet at ca. δ 8.10 ppm possibly overlapping with a doublet along with three additional doublets with a total of 5 different hydrogen signals. The ^13^C NMR spectrum also showed only one carbonyl carbon, and the rest of the signals were consistent with the presence of a *C*_*3*_ axis.

Density functional theory (DFT)^17^ calculations indicated that the Δ*G*° between *anti*-trimer **7** and *syn*-trimer **7** is only 0.1 kcal/mol, which is within the margin of calculation error (Figure 2). Unlike the previous reported case with the presence of 5-membered rings,^18^ the DFT calculations suggest that the two isomers are essentially isoenergetic with the ratio of the isolated yields (8.5% vs 2.6%) close to the theoretical random ratio of 3:1. It also suggests that during macrocyclization the two *ortho* sides of the bromo substituents are essentially identical with little effects from the *meta*-substituted dimethylene linkages.

**Figure 2:**
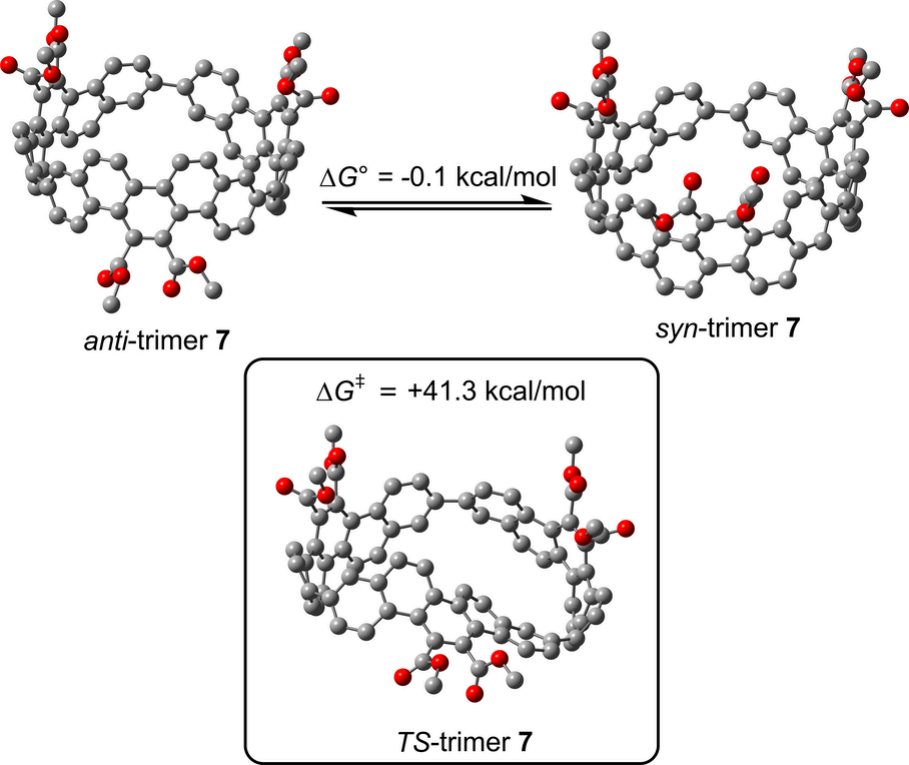
DFT-optimized structures of *anti*-trimer **7**, *syn*-trimer **7**, and the transition state (*TS*-trimer **7**) and their relative energies.

DFT calculations also show that the rotation barrier (Δ*G* ^‡^) from *anti*-trimer **7** to *syn*-trimer **7** is 41.3 kcal/mol, allowing the two isomers to be separated without equilibration as observed in the NMR spectra. The bending of the picene units and the single bonds connecting them together are responsible for the high rotational barrier. It was previously reported that the rotational barrier was high in a [6]­CPP derivative bearing three 9,9-dimethyl-9*H*-fluorene-2,7-diyl units.^19^

In summary, we successfully synthesized novel carbon nanohoops containing three picene units by employing a Ni­(cod)_2_/bpy-mediated macrocyclization reaction and a subsequent DDQ-promoted oxidation step. The key to this approach was the design of a dimethylene-linked substrate **6**, which allowed efficient formation of picene units directly within the CPP framework. The reaction produced a pair of *anti*-trimer **7** and *syn*-trimer **7** as isomers, offering valuable insights into the stereochemical dynamics of carbon nanohoop macrocyclization. This work underscores the versatility of the Diels–Alder reaction in producing precursor **6** having the two bromophenyl groups *cis* to each other in the central 6-membered diene system. The strategy of using Ni­(cod)_2_/bpy for macrocyclization^20^ followed by direct oxidation with DDQ provides innovative designs for the synthesis of complex CPP architectures. The incorporation of picene units opens new opportunities for exploring the optoelectronic properties of these carbon nanohoops and paves the way for their integration into advanced materials and armchair carbon nanobelt synthesis,^21^ which contains at least two bonds between the adjacent benzene rings. Future efforts will focus on refining these methodologies for carbon nanohoop and nanobelt syntheses and additional functionalization of these unique carbon nanohoops for diverse applications in nanotechnology, electronics, and photonics.

## Supplementary Material



## Data Availability

The data underling this study are available in the published article and its online Supporting Information.

## Notes

Experimental procedures, characterization data, single crystal X-ray crystallographic structure; NMR spectra for new compounds; computational coordinates and energetic details of DFT calculations (PDF)
