# Supplementary material for: Synthesis and Structures of Carbon Nanohoops Containing Three Picene Units with Each Having Two Carbomethoxy Substituents
Source: Org Lett. 2025 Jun 17;27(25):6578–82. doi: 10.1021/acs.orglett.5c01384 (PMC12210269; doi:10.1021/acs.orglett.5c01384)
Supplement: Supplementary file 1 [file ol5c01384_si_001.pdf]

Synthesis and Structures of Carbon Nanohoops Containing Three Picene Units with Each Having Two Carbomethoxy Substituents

Liu Li, Stephen M. Long, Nathaniel J. Selvaraj, Brian S. Dolinar, Brian V. Popp,

and Kung K. Wang*

C. Eugene Bennett Department of Chemistry, West Virginia University

Morgantown, West Virginia 26506, United States

Supporting Information

Table of Contents

Page

S3	General Experimental Methods
S3−S4	Experimental Procedure for Diketone **2**.
S4	Experimental Procedure for Diol **3**.
S4	Experimental Procedure for Diene **4**.
S5	Experimental Procedure for Hexahydropicene **6**.
S5−S6	Experimental Procedure for *anti*-Trimer **7** and *syn*-Trimer **7**.
S7	Figure S1. Thermal Ellipsoid Plot of the Crystal Structure of Hexahydropicene **6**.
S8−S9	Description of the X-ray Structural Analysis of Hexahydropicene **6**.
S10	References Cited
S11−S12	^{1}H and ^{13}C NMR Spectra of Diketone **2.**

S13−S14 ^{1}H and ^{13}C NMR Spectra of Diene **4.**

S15−S16 ^{1}H and ^{13}C NMR Spectra of Hexahydropicene **6**.

S17−S19 ^{1}H and ^{13}C NMR Spectra of *anti*-Trimer **7**.

S20−S21 ^{1}H and ^{13}C NMR Spectra of *syn*-Trimer **7**.

S22 Figure S2. HRMS Spectra of *anti*-Trimer **7** and *syn*-Trimer **7**.

S23 Computational Details and References.

S24−S35 Computational Coordinates and Energetic Details.

General Experimental Methods.

All reactions were conducted in oven-dried (120 °C) glassware under a nitrogen atmosphere. An oil bath was used as the heat source for the reactions that required heating. Chemicals, including 6-bromo-1-tetralone (**1**), Ni(cod)$_2$, 2,2'-bipyridyl, 2,3-dichloro-5,6-dicyano-1,4-benzoquinone (DDQ), and lithium diisopropylamide (2.0 M in THF), were purchased from chemical suppliers and were used as received. Chemical shifts of NMR spectra were reported as parts per million (δ) relative to the signal of $CHCl_3$ at 7.26 ppm or DMSO-d_5 at 2.50 ppm or benzene-d_5 at 7.16 ppm for ^{1}H NMR spectra and center line signal of the $CDCl_3$ triplet at 77.0 ppm or DMSO-d_6 at 39.5 ppm for ^{13}C{^{1}H} NMR spectra. HRMS spectra were obtained on an Orbitrap mass analyzer coupled with electrospray ionization (ESI).

Experimental Procedure for Diketone **2**. A solution of 6-bromo-1-tetralone (**1**) (2.5 g, 11.1 mmol) in dry THF (14 mL) was added dropwise to a flask containing lithium diisopropylamide (LDA, 5.8 mL of a 2 M solution, 11.6 mmol) in THF at –78 °C under a nitrogen atmosphere. The reaction was stirred for 30 mins at –78 °C before a solution of $FeCl_3$ (1.8 g, 11.1 mmol) in DMF (14 mL) was added dropwise at –78 °C under a nitrogen atmosphere. The reaction mixture was allowed to warm to rt and was further stirred for 12 h before it was quenched with 200 mL of a 1 M aqueous HCl solution. After 30 min of stirring, the reaction mixture was filtered and washed sequentially with water, ethanol, and hexanes to provide diketone **2** (2.0 g, 4.46 mmol, 80% yield) as a white solid: ^{1}H NMR ($CDCl_3$, 400 MHz) δ 7.92 and 7.88 (2 H, two doublets from the two diastereomers, J = 8.0, 8.8 Hz), 7.49–7.42 (m, 4 H), 3.55 (m, 1 H), 3.27–2.85 (m, 5 H), 2.24–1.91 (m, 4 H); ^{13}C{^{1}H} NMR ($CDCl_3$, 100 MHz) δ 197.9, 196.6, 145.9, 145.3, 131.7, 131.47, 131.42, 131.40, 130.2, 130.1, 129.4, 129.1, 128.6, 128.4, 49.2, 47.8, 29.4, 29.2, 25.4, 25.2; HRMS (ESI)

m/z [M + Na]$^+$ calcd for $C_{20}H_{16}Br_2O_2Na$ 468.9409, 470.9389, 472.9368; found 468.9406, 470.9385, 472.9367.

Experimental Procedures for Diol **3** *and Diene* **4**. To a 200 mL flask were added diketone **2** (1.0 g, 2.23 mmol) and sodium borohydride (768 mg, 20.3 mmol). The flask was flushed with nitrogen before 45 mL of THF was introduced via cannula. The reaction mixture was heated at reflux for 1 h before 2.7 mL of methanol was added dropwise. The reaction mixture was heated at reflux for an additional 12 h before it was cooled to rt. Then the reaction mixture was quenched with 50 mL of a 3.0 M sodium hydroxide solution. After 10 min of stirring, the reaction mixture was filtered and then washed with 20 mL of a 3.0 M sodium hydroxide solution and water (20 mL). After the product was air-dried, the crude diol **3** (900 mg) was obtained as a white solid, which was used without further purification.

To a flask containing 900 mg of the previously obtained diol **3** in 10 mL of pyridine was added PBr_3 (2.15 g, 7.96 mmol), and the reaction mixture was heated at 70 °C for 2 h before it was allowed to cool to rt. An aqueous solution of NaOH (3 M, 20 mL) was then added to quench the reaction mixture. The resulting precipitate was filtered, washed by water, and air dried to give diene **4** as a yellow solid (510 mg, 1.23 mmol, 55% yield over two steps): ^{1}H NMR ($CDCl_3$, 400 MHz) δ 7.30–7.27 (m, 4 H), 6.95 (d, J = 8.6 Hz, 2 H), 6.66 (s, 2 H), 2.87 (t, J = 8.1 Hz, 4 H), 2.63 (t, J = 8.1 Hz, 4 H); ^{13}C{^{1}H} NMR ($CDCl_3$, 100 MHz) δ 138.4, 137.5, 133.6, 130.1, 129.5, 128.0, 122.7, 120.3, 27.8, 24.0; HRMS (ESI) *m/z* [M + H]$^+$ calcd for $C_{20}H_{17}Br_2$ 414.9692, 416.9671, 418.9651; found 414.9683, 416.9657, 418.9640.

Experimental Procedure for Hexahydropicene **6**. To a mixture of diene **4** (330 mg, 0.79 mmol) and dimethyl acetylenedicarboxylate (**5**, 563 mg, 3.96 mmol) was added dry toluene (3 mL) under a nitrogen atmosphere. The reaction mixture was stirred at 90 °C for 16 h before it was allowed to cool to rt. After removal of solvent in vacuo, the residue was purified by flash column chromatography (silica gel/ethyl acetate:hexanes = 1:9) to provide hexahydropicene **6** as a white solid (261 mg, 0.47 mmol, 59% yield): ^{1}H NMR ($CDCl_3$, 400 MHz) δ 7.22 (d, J = 6.2 Hz, 2 H), 7.21 (s, 2 H), 7.01 (d, J = 8.6 Hz, 2 H), 4.27 (s, 2 H), 3.81 (s, 6 H), 2.90–2.70 (m, 4 H), 2.49–2.42 (m, 2 H), 2.12–2.00 (m, 2 H); $^{13}C\{^{1}H\}$ NMR ($CDCl_3$, 100 MHz) δ 168.1, 140.6, 139.1, 136.8, 129.9, 129.2, 125.5, 125.1, 119.8, 52.7, 42.6, 27.9, 25.6; HRMS (ESI) *m/z* $[M + Na]^+$ calcd for $C_{26}H_{22}Br_2O_4Na$ 578.9777, 580.9757, 582.9736; found 578.9796, 580.9774, 582.9750.

Recrystallization of **6** from a mixture of hexanes and dichloromethane produced a single crystal suitable for an X-ray structure analysis.

Experimental Procedure for anti-Trimer **7** *and syn-Trimer* **7**. To a 500 mL flask were added hexahydropicene **6** (200 mg, 0.358 mmol) and 2,2′-bipyridyl (84 mg, 0.537 mmol). The flask was flushed with nitrogen and placed in a glovebox under a nitrogen atmosphere before $Ni(cod)_2$ (148 mg, 0.537 mmol) and anhydrous THF (36 mL) were added. The flask was fitted with a condenser and a rubber septum and then removed from the glovebox. The reaction mixture was heated at reflux for 16 h before it was allowed to cool to rt. Then the reaction mixture was passed through a short pad of silica gel column (4 cm) and eluted with a mixture of ethyl acetate and dichloromethane (1:1). The combined eluates were concentrated and dried in vacuo. The residue was treated with DDQ (406 mg, 1.79 mmol) in 4 mL of chlorobenzene at 120 °C for 3 h. The reaction mixture was

then passed through a short basic aluminum oxide column and eluted with 150 mL of a mixture of dichloromethane and ethyl acetate (1:1). The combined eluates were concentrated in vacuo, and the residue was purified by flash column chromatography (silica gel/dichloromethane:ethyl acetate = 95:5) to produce *anti*-trimer **7** (12 mg, 0.0102 mmol, 8.5% yield) and *syn*-trimer **7** (3.6 mg, 0.00306 mmol, 2.6% yield) as yellow solids. *anti*-trimer **7**: ^{1}H NMR (DMSO-d_6, 400 MHz) δ 8.60–8.51 (m, 8 H), 8.44–8.42 (br m, 4 H), 8.22 (d, J = 9.2 Hz, 4 H), 8.15–8.07 (m, 8 H), 8.06–8.00 (m, 6 H), 3.93 (s, 6 H), 3.92 (s, 6 H), 3.90 (s, 6 H); ^{13}C{^{1}H} NMR (DMSO-d_6, 100 MHz) δ 168.29, 168.26, 168.1, 133.75, 133.72, 133.70, 133.0, 132.9, 130.6, 130.5, 130.4, 130.2, 129.1, 128.97, 128.93, 127.2, 127.0, 126.7, 126.6, 126.2, 125.8, 125.7, 125.4, 123.7, 123.6, 121.0, 53.2; HRMS (ESI) *m/z* [M]$^+$ calcd for $C_{78}H_{48}O_{12}$ 1176.3140; found 1176.3121. *syn*-trimer **7**: ^{1}H NMR (DMSO-d_6, 400 MHz) δ 8.58–8.52 (m, 12 H), 8.13–8.06 (m, 12 H), 8.03 (d, J = 9.5 Hz, 6 H), 3.90 (s, 18 H); ^{13}C{^{1}H} NMR (DMSO-d_6, 100 MHz) δ 168.2, 133.7, 132.9, 130.5, 130.2, 129.1, 126.9, 126.5, 125.8, 123.7, 120.9, 118.6, 53.2; HRMS (ESI) *m/z* [M]$^+$ calcd for $C_{78}H_{48}O_{12}$ 1176.3140; found 1176.3152.

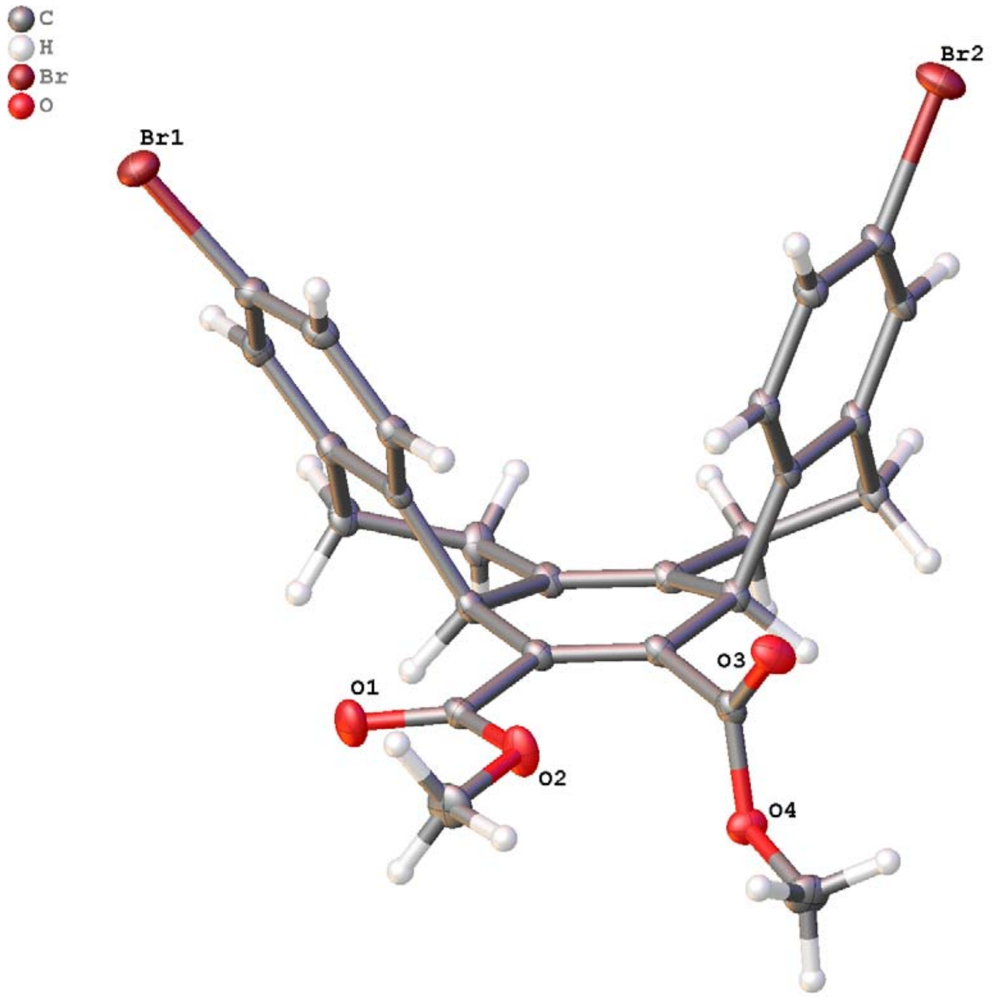

Figure S1. Thermal Ellipsoid Plot of the Crystal Structure of Hexahydropicene **6**. All Non-hydrogen Atoms Are Drawn As 50% Thermal Probability Ellipsoids.

Description of the X-ray Structural Analysis of Hexahydropicene **6**.

A colorless block-shaped crystal measuring 0.452 mm x 0.364 mm x 0.280 mm was selected under polybutene oil using a MiTeGen Micromount and mounted at 100(1) K to a D8-Venture diffractometer equipped with a Mo sealed tube X-ray source, a Triumph monochromator, and a Photon 2 CMOS area detector. The unit cell was determined from reflections harvested with a signal to noise ratio (I/σ) of at least 10 from a series of 2 ω scans of 6° with 0.5° frames using APEX3.[S1] A complete set of data was collected to a resolution of 0.70 Å using 8 ω and 1 φ scans. The data were integrated using SAINT and corrected for absorption using SADABS.[S2] The systematic absences and E-statistics of the data were uniquely consistent with the space group $P2_1/c$. The structure was solved using the intrinsic phasing routine of SHELXT.[S3] The non-hydrogen atoms were located from a Fourier difference map of the electron density and anisotropically refined using the least-squares algorithm of SHELXL.[S4] The hydrogen atoms were then placed in calculated positions and refined with riding thermal parameters.

The angle between bromophenyl rings Br1 and Br2 is 54.18(13)°.

The final structure consisted of 291 parameters refined against 6808 independent reflections, giving refinement residuals of R_1 = 0.0299 (based off F^2 for $I > 2\sigma$) and wR_2 = 0.0821 (based of F^2 for all reflections). The final difference Fourier map was featureless.

Table S1. Crystal data and structure refinement for Hexahydropicene 6.

Identification code	kw49_0m_a
Empirical formula	$C_{26}H_{22}O_4Br_2$
Formula weight	558.25
Temperature/K	100.00
Crystal system	monoclinic
Space group	$P2_1/c$
a/Å	15.8508(3)
b/Å	7.8073(2)
c/Å	18.2706(4)
α/°	90
β/°	100.9880(10)
γ/°	90
Volume/Å^3	2219.57(9)
Z	4
ρ_{calc}g/cm^3	1.671
μ/mm^{-1}	3.684
F(000)	1120.0
Crystal size/mm^3	0.452 × 0.364 × 0.28
Radiation	Mo Kα (λ = 0.71073)
2Θ range for data collection/°	5.236 to 61.112
Index ranges	$-22 \le h \le 22$, $-11 \le k \le 11$, $-26 \le l \le 26$
Reflections collected	143664
Independent reflections	6808 [R_{int} = 0.0491, R_{sigma} = 0.0163]
Data/restraints/parameters	6808/0/291
Goodness-of-fit on F^2	1.064
Final R indexes [I>=2σ (I)]	R_1 = 0.0299, wR_2 = 0.0792
Final R indexes [all data]	R_1 = 0.0353, wR_2 = 0.0821
Largest diff. peak/hole / e Å^{-3}	1.28/-1.53

References Cited

S1 Bruker-AXS (2016). APEX 3 version 2016.9-0. Madison, Wisconsin, USA

S2. (a) Bruker-AXS (2015). *SAINT V8.37A.* Madison, Wisconsin, USA. (b) Bruker-AXS (2014). *SADABS*, Madison, Wisconsin, USA.

S3. Sheldrick, G. M. *Acta Crystallogr. A* **2015**, *A71*, 3-8.

S4. (a) Sheldrick, G. M. *Acta Crystallogr. C* **2015**, *C71*, 3-8.; (b) Dolomanov, O. V.; Bourhis, L. J.; Gildea, R. J.; Howard, J. A. K.; Puschmann, H. *J. Appl. Crystallogr.* **2009**, *42*, 339-341.

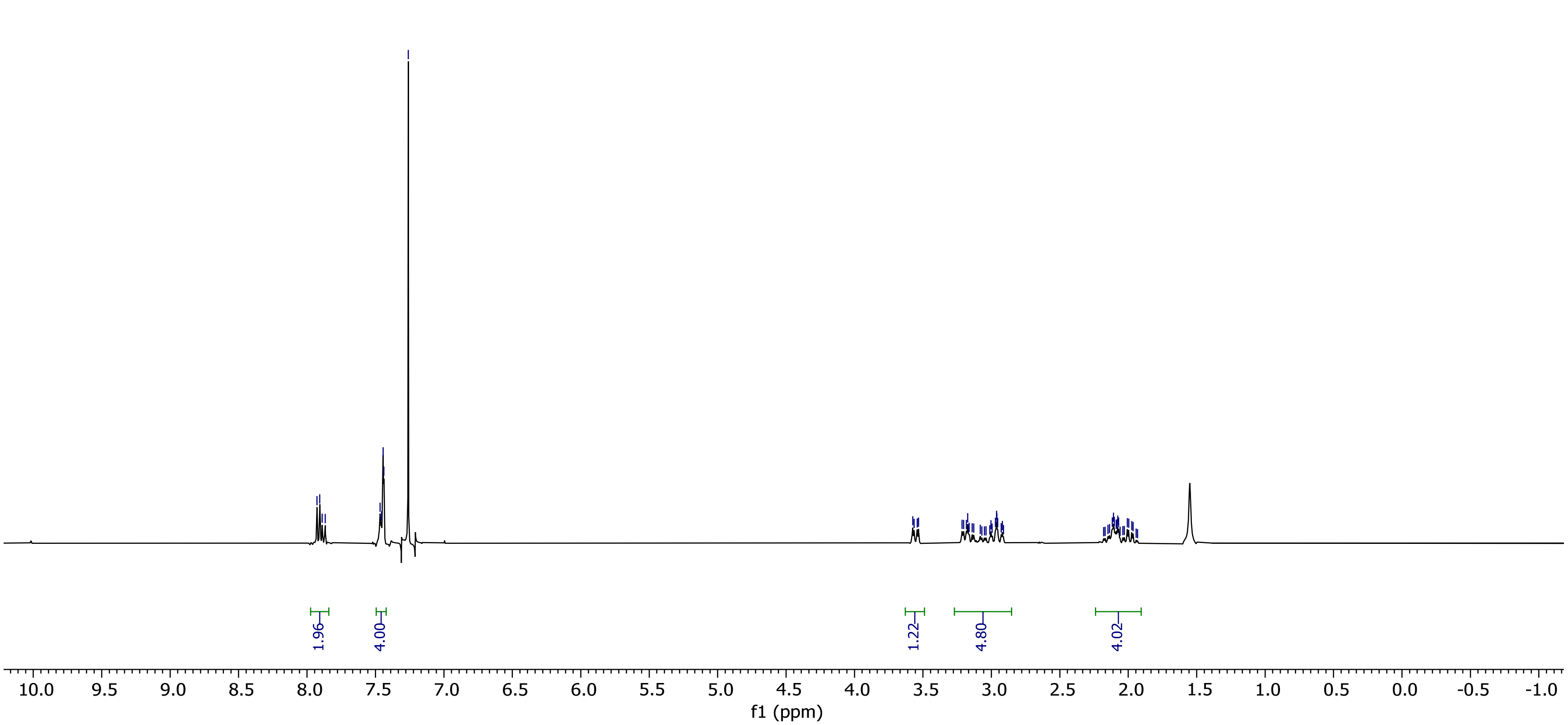

7.9273
7.9072
7.8891
7.8671
7.4665
7.4552
7.4435
7.4384
7.2600
3.5743
3.5643
3.5418
3.5320
3.2141
3.2026
3.1828
3.1735
3.1620
3.1405
3.1292
3.0815
3.0695
3.0509
3.0394
3.0115
3.0035
2.9944
2.9709
2.9621
2.9597
2.9535
2.9283
2.9200
2.9117
2.1796
2.1690
2.1480
2.1370
2.1167
2.1083
2.1020
2.0878
2.0809
2.0765
2.0699
2.0594
2.0393
2.0291
2.0066
1.9965
1.9740
1.9645
1.9424
1.9319
1.96
4.00
1.22
4.80
4.02
10.0 9.5 9.0 8.5 8.0 7.5 7.0 6.5 6.0 5.5 5.0 4.5 4.0 3.5 3.0 2.5 2.0 1.5 1.0 0.5 0.0 -0.5 -1.0
f1 (ppm)

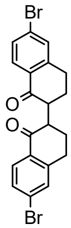

Br
O
O
Br

2

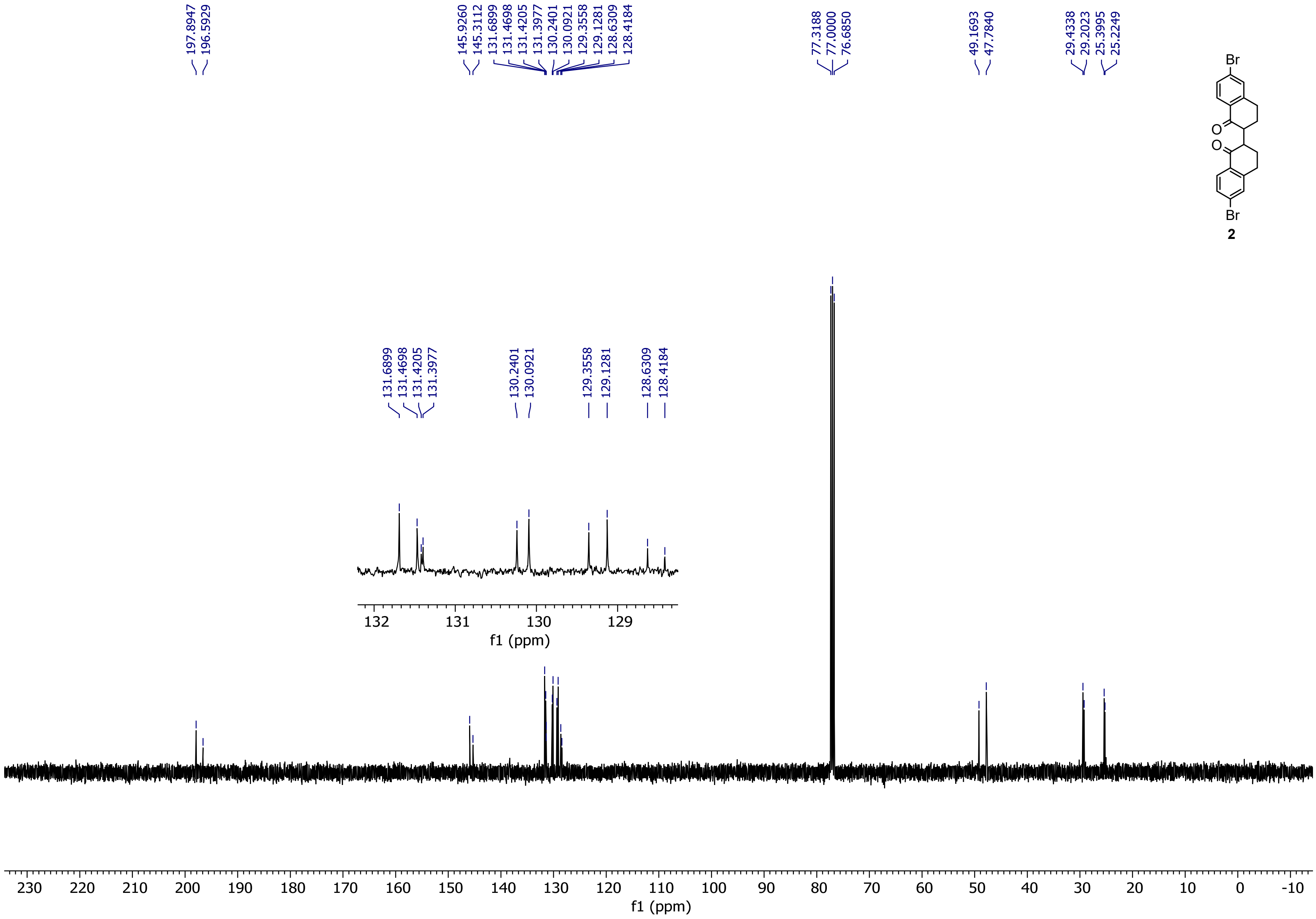
197.8947
196.5929
145.9260
145.3112
131.6899
131.4698
131.4205
131.3977
130.2401
130.0921
129.3558
129.1281
128.6309
128.4184
77.3188
77.0000
76.6850
49.1693
47.7840
29.4338
29.2023
25.3995
25.2249
f1 (ppm)
f1 (ppm)
Br
O
O
Br

2

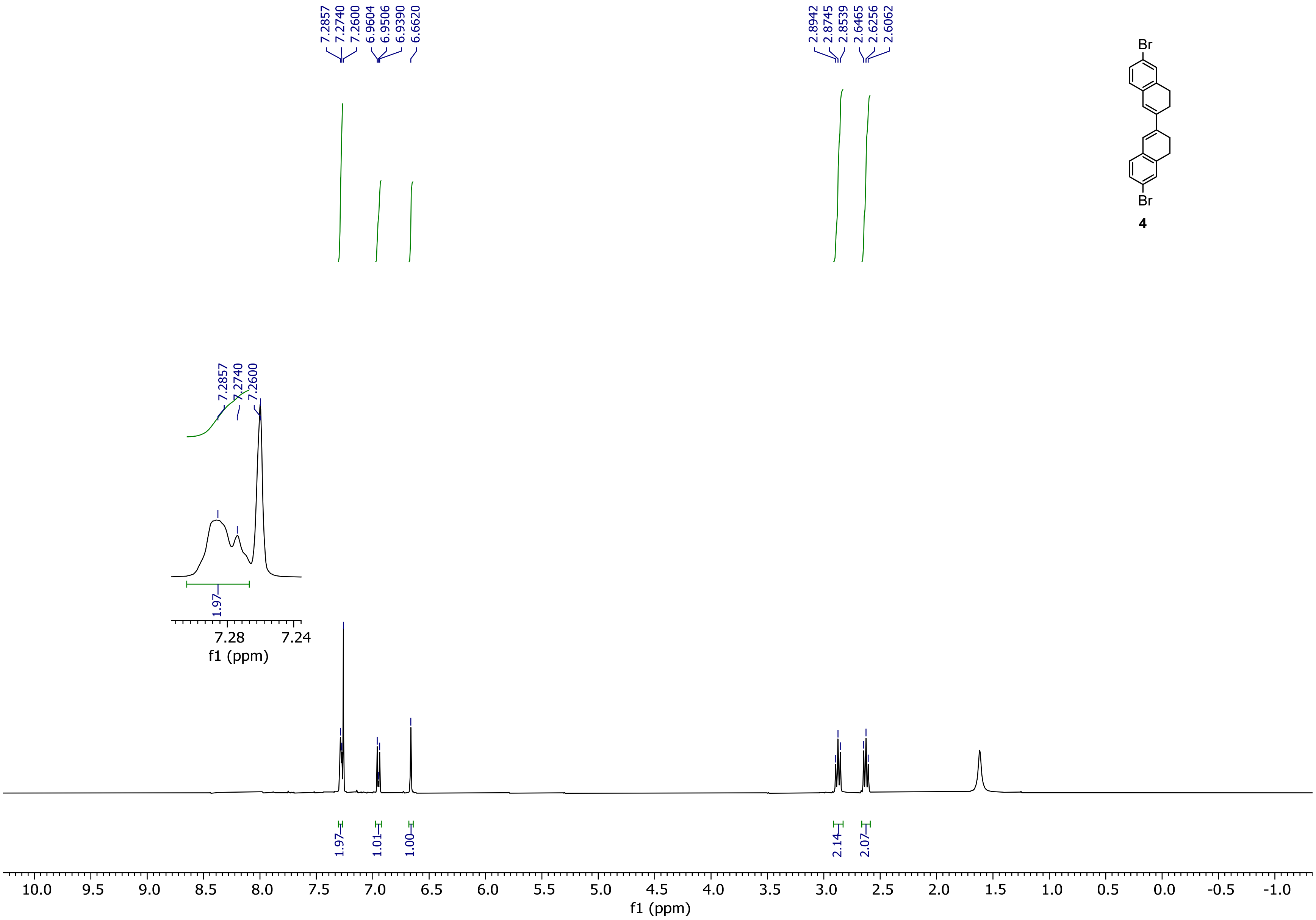
7.2857
7.2740
7.2600
6.9604
6.9506
6.9390
6.6620
2.8942
2.8745
2.8539
2.6465
2.6256
2.6062
1.97
1.01
1.00
2.14
2.07
7.28
7.24
f1 (ppm)
10.0 9.5 9.0 8.5 8.0 7.5 7.0 6.5 6.0 5.5 5.0 4.5 4.0 3.5 3.0 2.5 2.0 1.5 1.0 0.5 0.0 -0.5 -1.0
f1 (ppm)
Br
Br

4

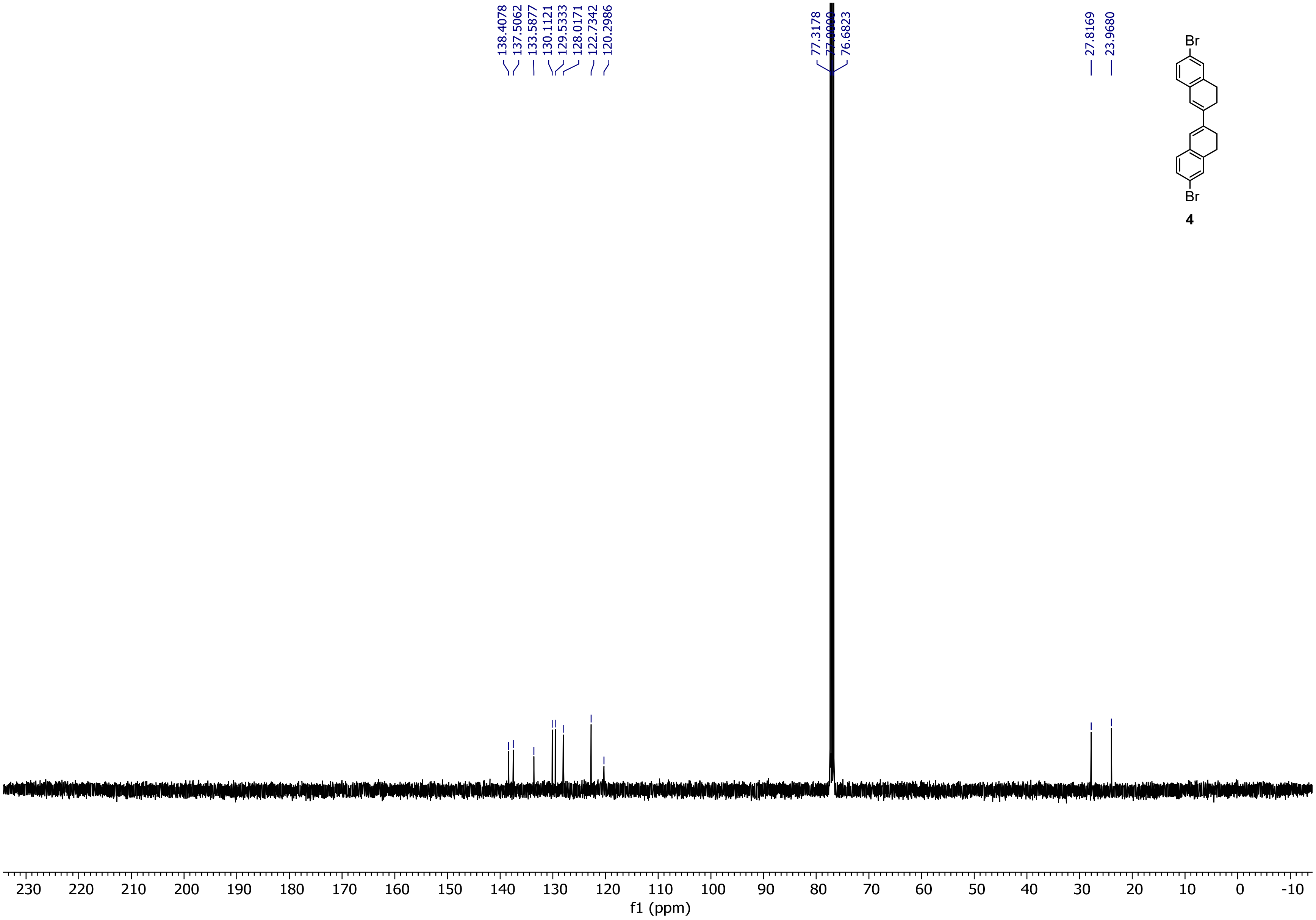
138.4078
137.5062
133.5877
130.1121
129.5333
128.0171
122.7342
120.2986
77.3178
76.6823
27.8169
23.9680
230 220 210 200 190 180 170 160 150 140 130 120 110 100 90 80 70 60 50 40 30 20 10 0 -10
f1 (ppm)
Br
Br

4

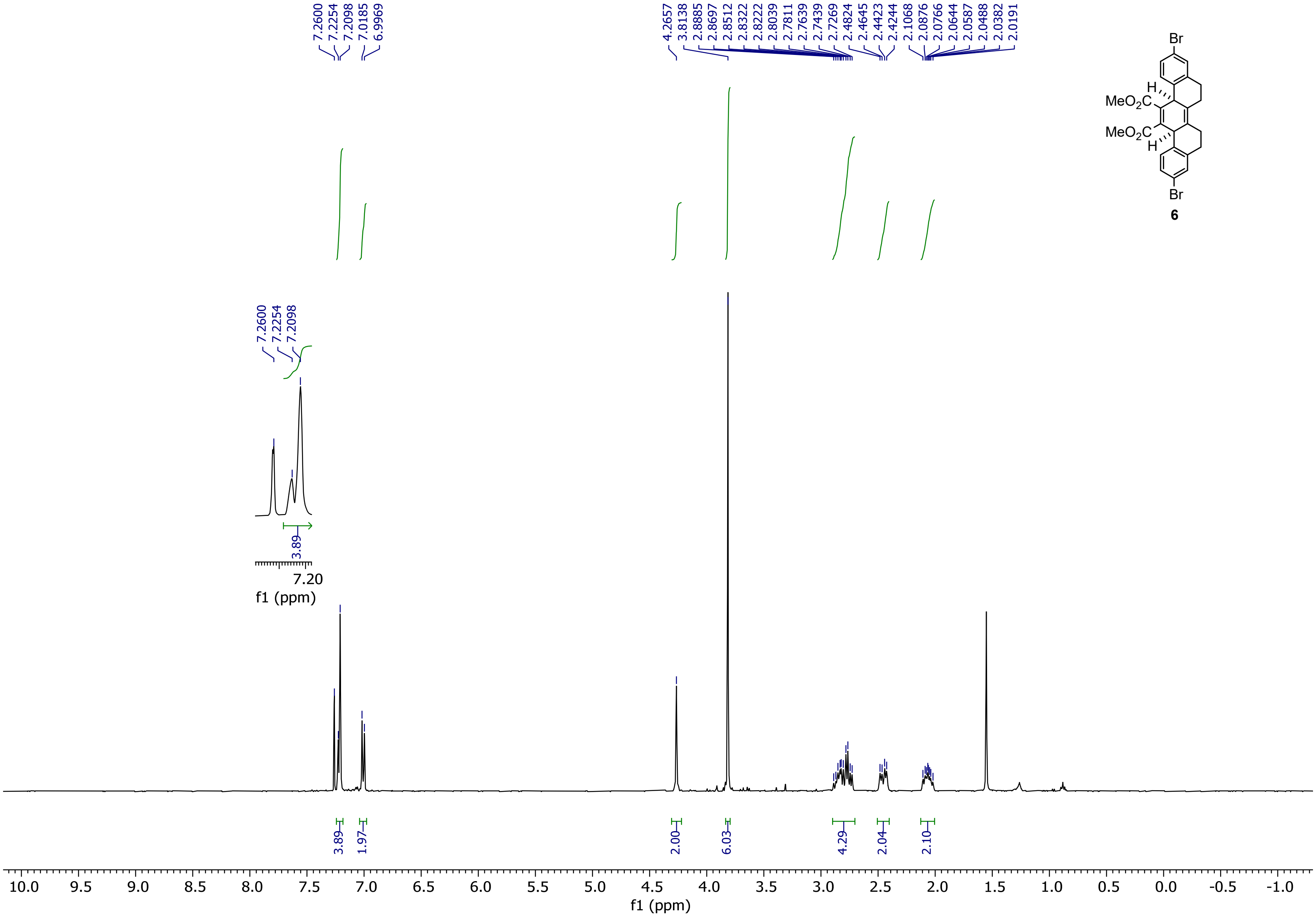
7.2600
7.2254
7.2098
7.0185
6.9969
4.2657
3.8138
2.8885
2.8697
2.8512
2.8322
2.8222
2.8039
2.7811
2.7639
2.7439
2.7269
2.4824
2.4645
2.4423
2.4244
2.1068
2.0876
2.0766
2.0644
2.0587
2.0488
2.0382
2.0191
3.89
1.97
2.00
6.03
4.29
2.04
2.10
7.20
f1 (ppm)
f1 (ppm)
Br
H
MeO2C
MeO2C
H
Br

6

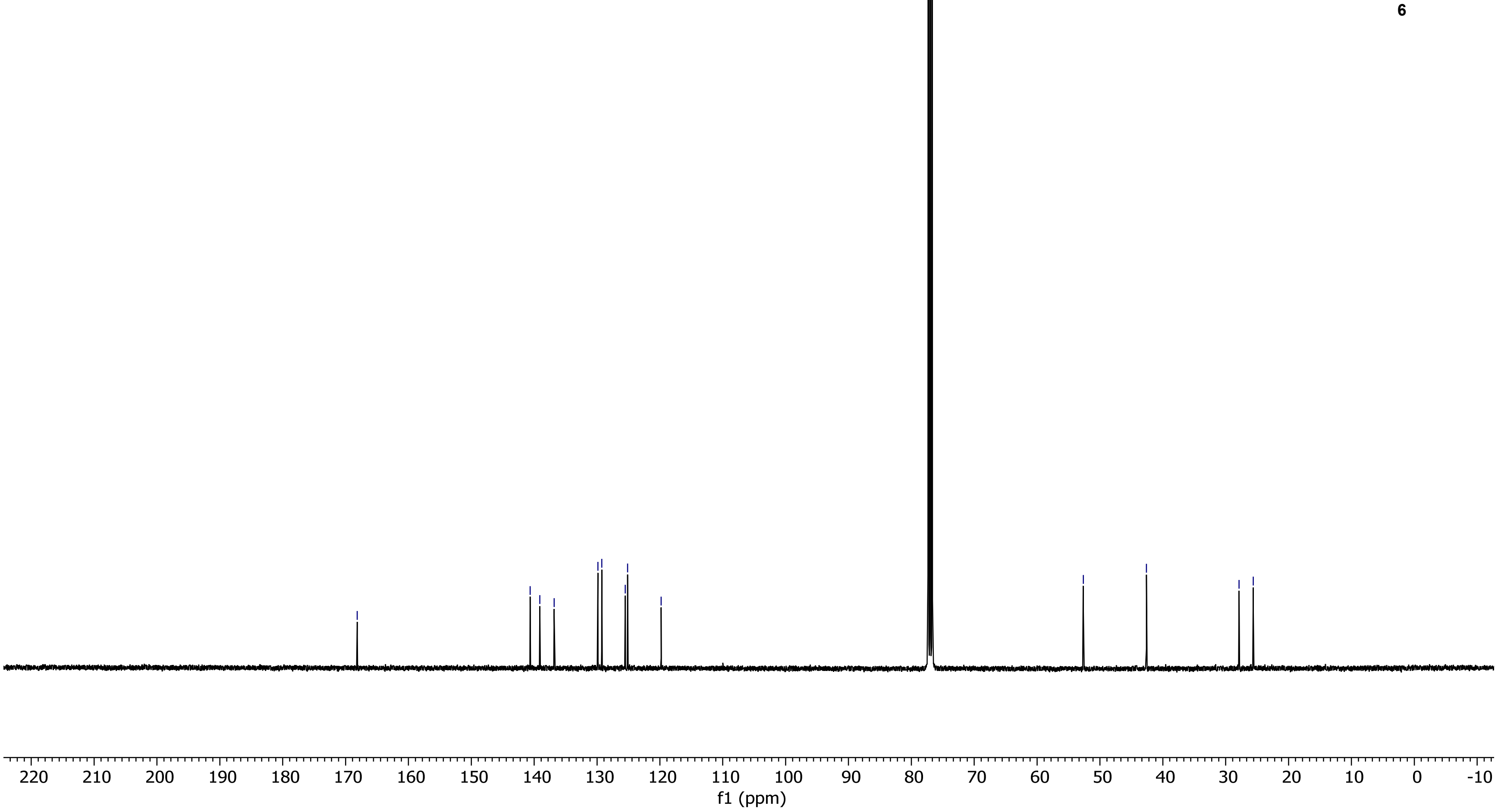
168.1456
140.6310
139.1032
136.8149
129.8619
129.2425
125.5030
125.1444
119.7963
77.3178
76.6821
52.6539
42.6048
27.8843
25.6144
220 210 200 190 180 170 160 150 140 130 120 110 100 90 80 70 60 50 40 30 20 10 0 -10
f1 (ppm)

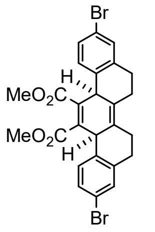
Br
H
MeO$_2$C
MeO$_2$C
H
Br
6

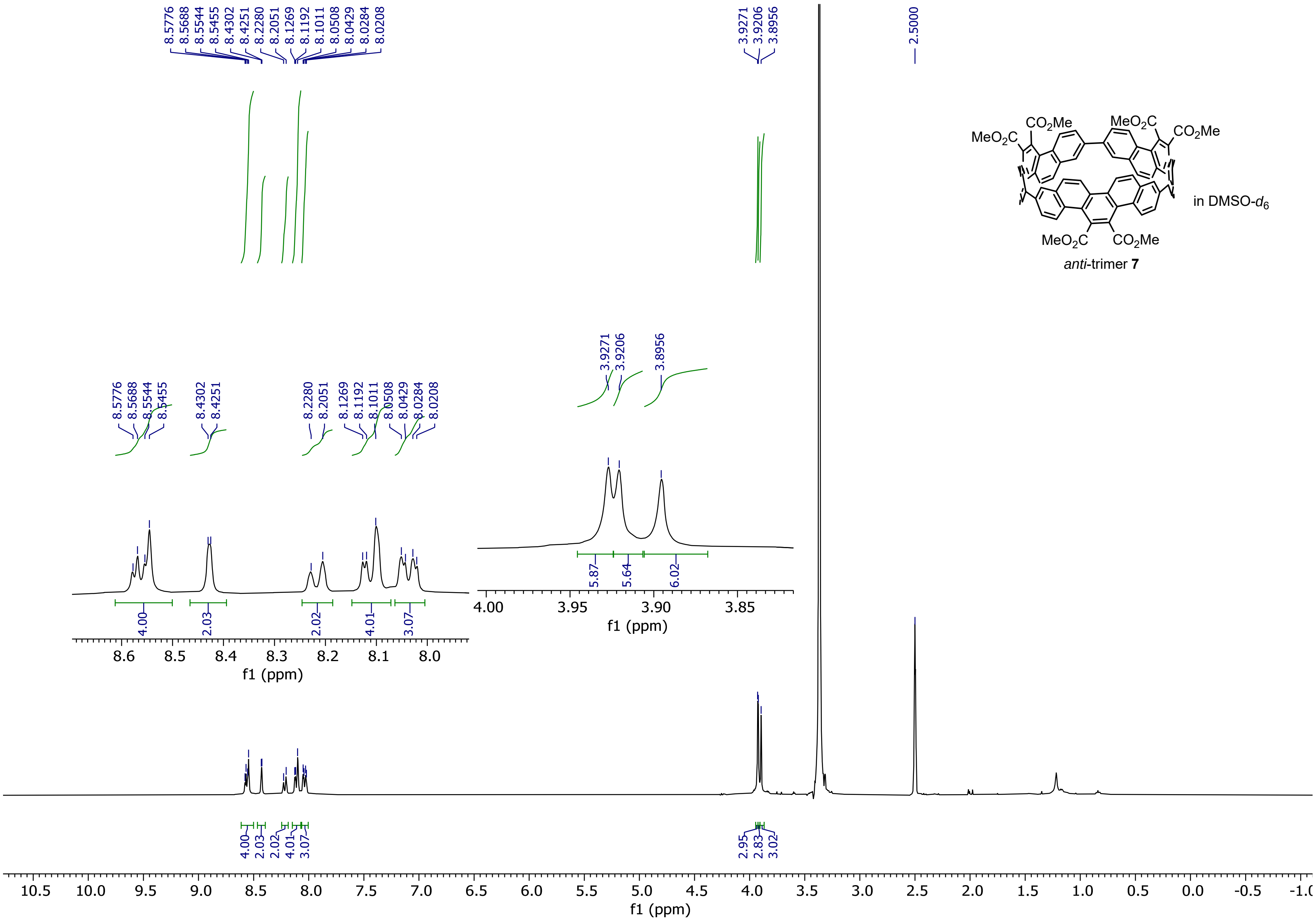
8.5776
8.5688
8.5544
8.5455
8.4302
8.4251
8.2280
8.2051
8.1269
8.1192
8.1011
8.0508
8.0429
8.0284
8.0208
3.9271
3.9206
3.8956
2.5000
4.00
2.03
2.02
4.01
3.07
5.87
5.64
6.02
2.95
2.83
3.02
f1 (ppm)
MeO2C
CO2Me
in DMSO-d6
anti-trimer 7

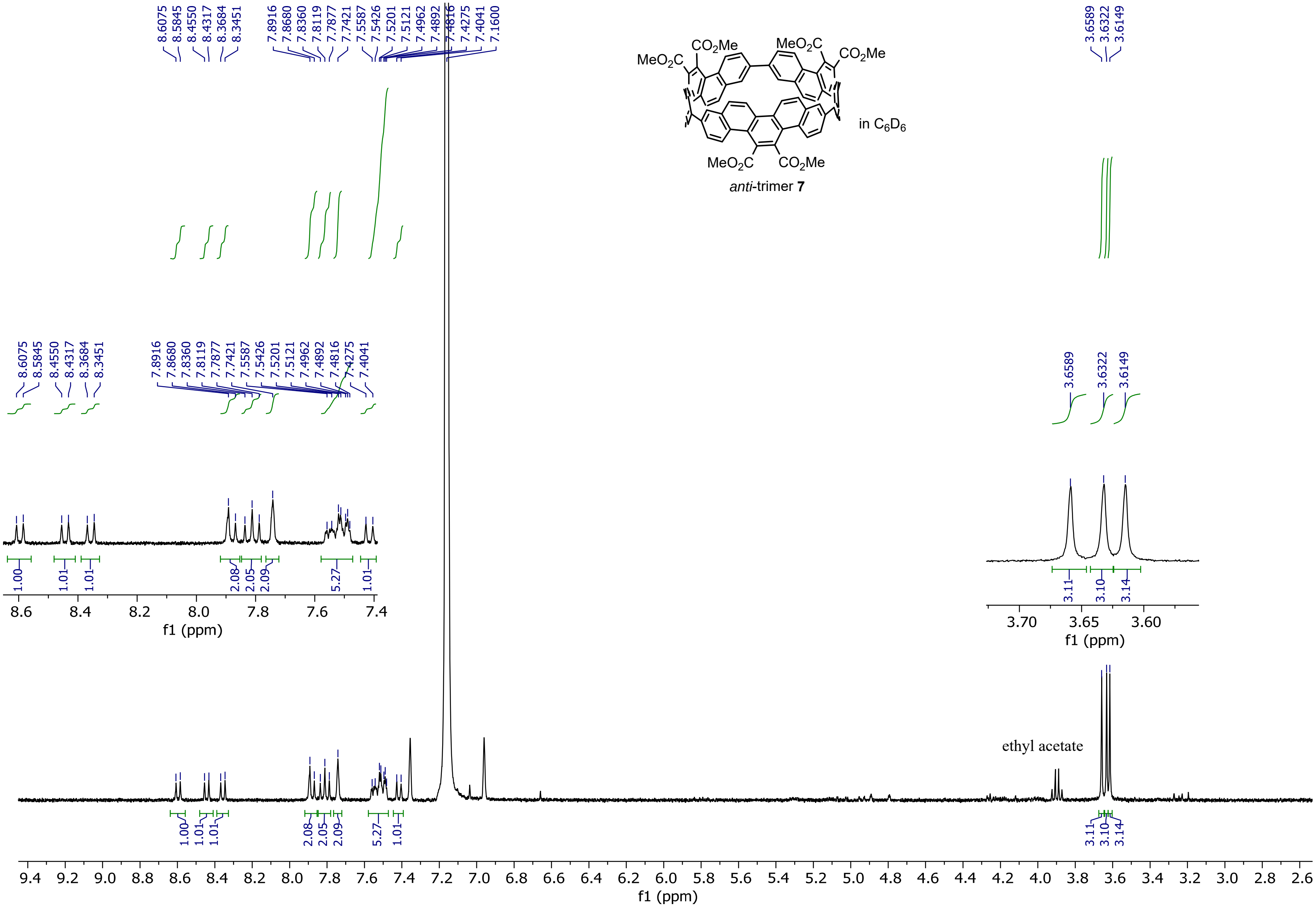
MeO2C
CO2Me
MeO2C
CO2Me
MeO2C
CO2Me
in C6D6
anti-trimer 7
ethyl acetate
f1 (ppm)

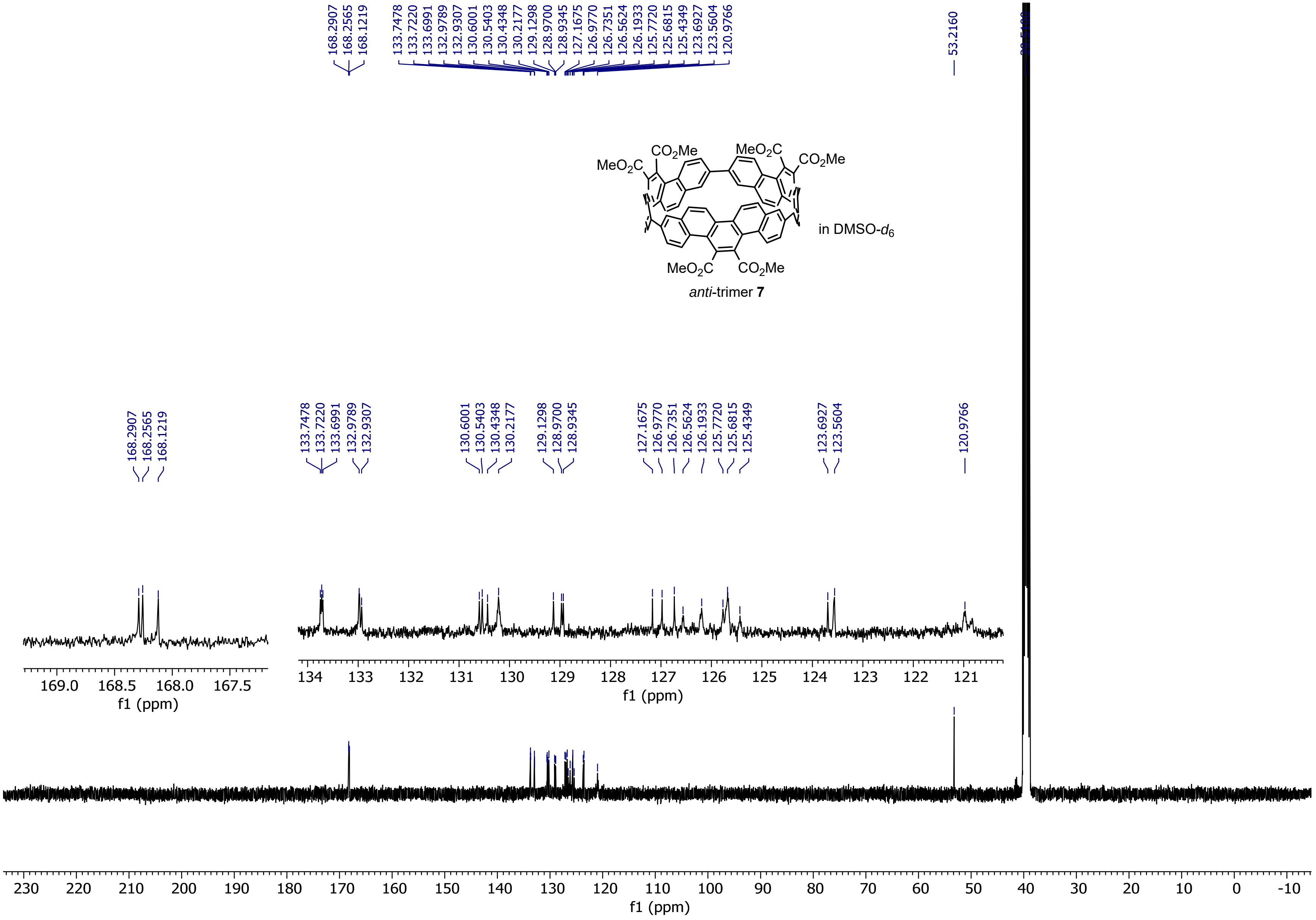
168.2907
168.2565
168.1219
133.7478
133.7220
133.6991
132.9789
132.9307
130.6001
130.5403
130.4348
130.2177
129.1298
128.9700
128.9345
127.1675
126.9770
126.7351
126.5624
126.1933
125.7720
125.6815
125.4349
123.6927
123.5604
120.9766
53.2160
MeO_2C
CO_2Me
MeO_2C
CO_2Me
MeO_2C
CO_2Me
in DMSO-d_6
anti-trimer **7**
f1 (ppm)
f1 (ppm)
f1 (ppm)

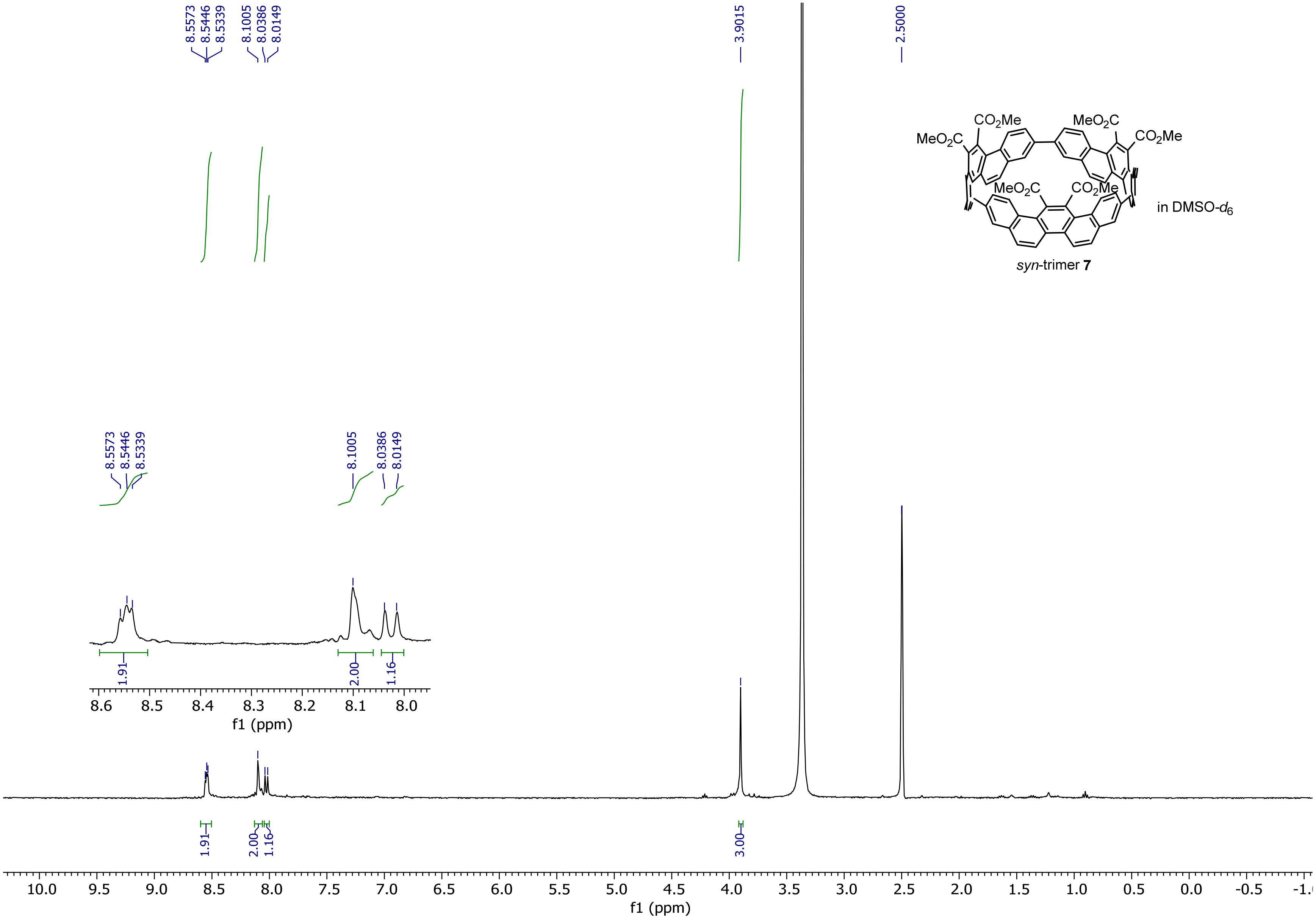
8.5573
8.5446
8.5339
8.1005
8.0386
8.0149
3.9015
2.5000
1.91
2.00
1.16
3.00
f1 (ppm)
CO2Me
MeO2C
in DMSO-d6
syn-trimer 7

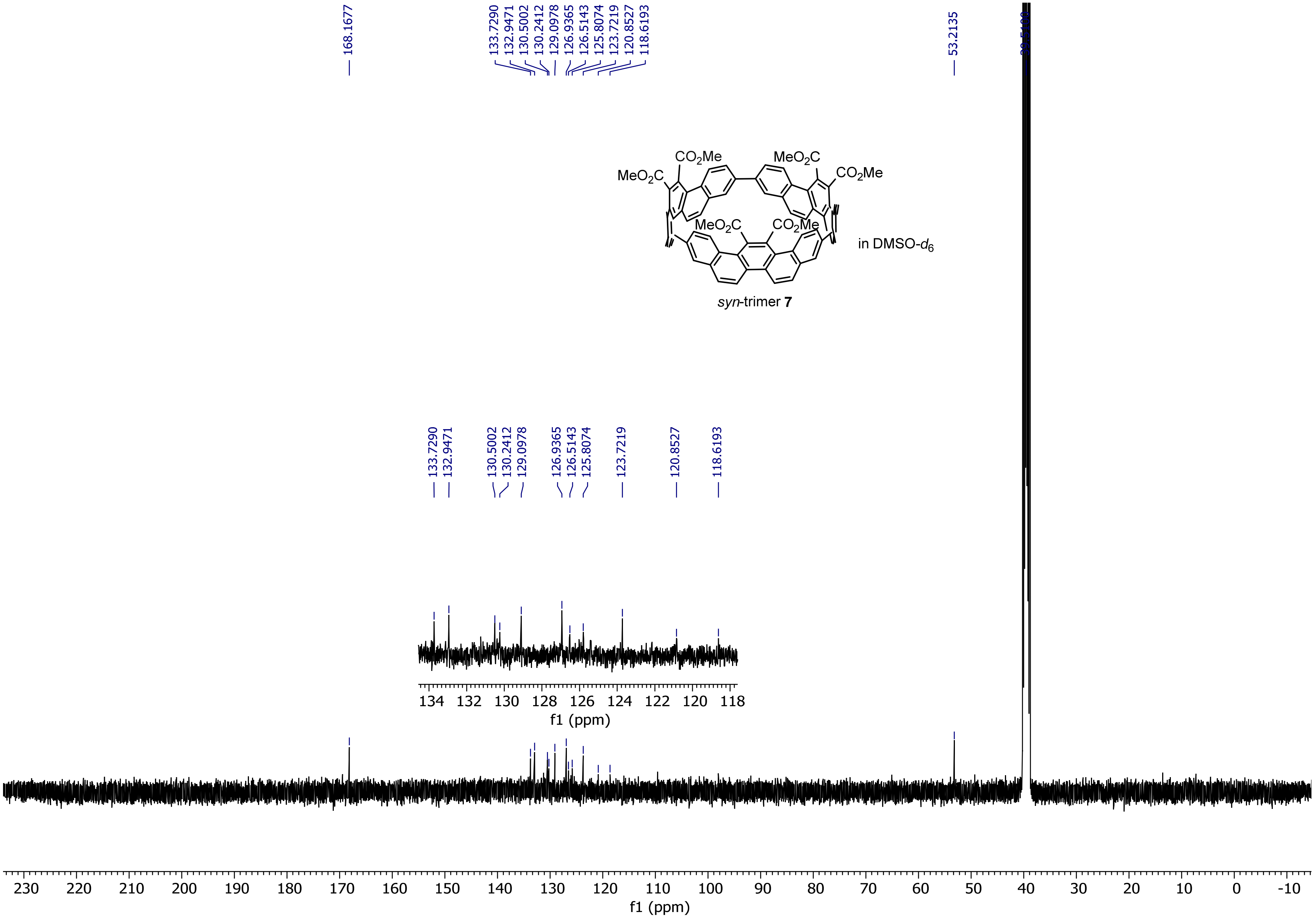
168.1677
133.7290
132.9471
130.5002
130.2412
129.0978
126.9365
126.5143
125.8074
123.7219
120.8527
118.6193
53.2135
CO2Me
MeO2C
MeO2C
CO2Me
MeO2C
CO2Me
in DMSO-d6
syn-trimer 7
f1 (ppm)
f1 (ppm)

Anti-Trimer 7

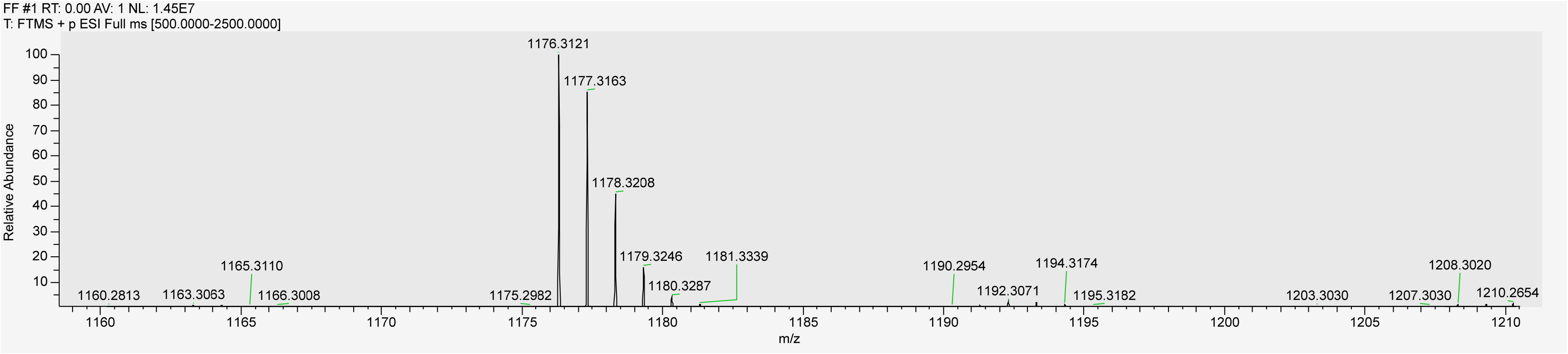

Syn-Trimer 7

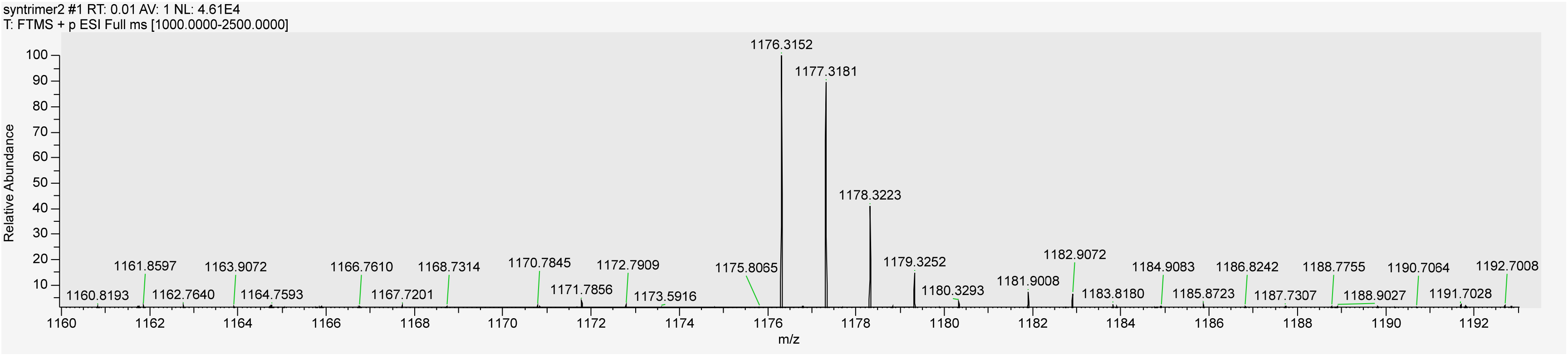

Figure S2. HRMS spectra of *anti*-trimer **7** and *syn*-trimer **7**.

Computational Details:

Spin-restricted density functional theory (RDFT) calculations were performed with the Gaussian 16 (G16) program.[S5] Geometry optimization and normal mode analyses were performed using the M06 functional and the 6-31G(d) basis set to identify ground state and transition state geometries.[S6] Transition state geometries were determined using generalized internal coordinates. The identified stationary points met or exceeded all default G16 convergence criteria including predicted changes in energy upon further optimization less than -1.0E-08 kcal/mol. Using the same functional-basis set, normal-modes were calculated for each optimized structure and thermochemical corrections were determined.

References:

S5 M. J. Frisch, G. W. Trucks, H. B. Schlegel, G. E. Scuseria, M. A. Robb, J. R. Cheeseman, G. Scalmani, V. Barone, G. A. Petersson, H. Nakatsuji, X. Li, M. Caricato, A. V. Marenich, J. Bloino, B. G. Janesko, R. Gomperts, B. Mennucci, H. P. Hratchian, J. V. Ortiz, A. F. Izmaylov, J. L. Sonnenberg, D. Williams-Young, F. Ding, F. Lipparini, F. Egidi, J. Goings, B. Peng, A. Petrone, T. Henderson, D. Ranasinghe, V. G. Zakrzewski, J. Gao, N. Rega, G. Zheng, W. Liang, M. Hada, M. Ehara, K. Toyota, R. Fukuda, J. Hasegawa, M. Ishida, T. Nakajima, Y. Honda, O. Kitao, H. Nakai, T. Vreven, K. Throssell, J. A. Montgomery, Jr., J. E. Peralta, F. Ogliaro, M. J. Bearpark, J. J. Heyd, E. N. Brothers, K. N. Kudin, V. N. Staroverov, T. A. Keith, R. Kobayashi, J. Normand, K. Raghavachari, A. P. Rendell, J. C. Burant, S. S. Iyengar, J. Tomasi, M. Cossi, J. M. Millam, M. Klene, C. Adamo, R. Cammi, J. W. Ochterski, R. L. Martin, K. Morokuma, O. Farkas, J. B. Foresman, and D. J. Fox, Gaussian, Inc., Wallingford CT, 2016.

S6 Zhao, Y.; Truhlar, D. G. Theor. Chem. Acc. 2006, 120, 215−241

Computational Coordinates and Energetic Details:

***Anti*-Trimer 7**

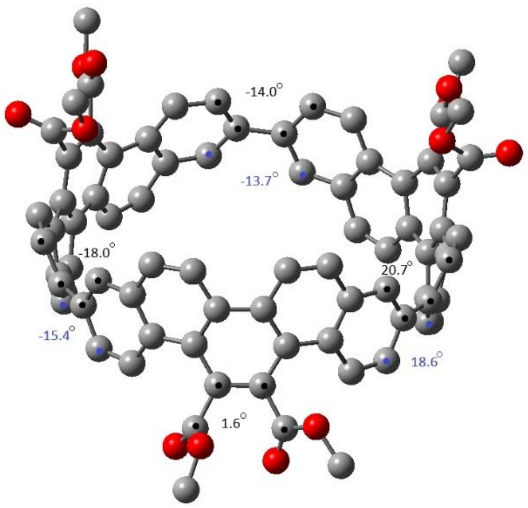

Calculation results at DFT-optimized geometry:

Total Energy: -2448041.498 kcal/mol

SCF Convergence: 0.30E-08

Enthalpy (298 K): -2447331.235 kcal/mol

Entropy (298 K): 379.138 eu

Free Energy (298 K): -2447444.216 kcal/mol

Three lowest frequencies: 8.77, 14.6, 17.2

Predicted change in Energy upon next optimization step: -8.976207E-09

C 4.42617200 -4.40860800 -0.98081300
C 3.61195500 -4.66044500 -2.09839000
C 4.16087300 -5.17479700 0.19934200
C 2.36184900 -5.35309100 -1.92752900
C 3.96280500 -4.04759200 -3.34490200
C 5.30728600 -3.25966200 -0.99679100
C 2.92742000 -5.76113500 0.39340500
C 5.24132600 -5.44357200 1.18980900
C 1.93159400 -5.75684000 -0.63762500
C 1.44966200 -5.43697600 -3.02263300
C 4.86298400 -3.03523900 -3.39730100
H 3.46484400 -4.35221800 -4.26079500
C 5.43770700 -2.51827600 -2.19797000
C 5.85139600 -2.66966700 0.16887800
C 2.69969400 -6.42960300 1.71842100
O 6.41917200 -5.60096500 0.56642600
O 5.10848400 -5.56328200 2.38794000
C 0.51717800 -6.01243000 -0.41935100
C 0.12372300 -5.61964200 -2.81177300
H 1.80100500 -5.26472300 -4.03531500
H 5.07485900 -2.53160500 -4.34035400
C 5.92582100 -1.20021200 -2.17149300
C 6.31298700 -1.37438800 0.17075800
H 5.80668900 -3.18653500 1.12555800
O 2.30325700 -5.53917700 2.63335900
O 2.84006900 -7.61040200 1.92037300
C 7.52960900 -5.81953100 1.42762400
C -0.38455600 -5.78463800 -1.49380800
C -0.09126900 -6.32349100 0.82354200
H -0.58110300 -5.58166500 -3.64231400
C 6.26326100 -0.56208500 -0.98874000
H 5.85900100 -0.63808600 -3.10215300
H 6.65253600 -0.95283700 1.11490800
C 2.20863400 -6.05376000 3.95630200
H 8.39224100 -5.95972300 0.77372000
H 7.68344000 -4.95062900 2.08015300
H 7.36838200 -6.70430400 2.05219800
C -1.75596300 -5.58334100 -1.25075600
C -1.43461600 -6.12423000 1.03802700
H 0.49131900 -6.69983800 1.65501700
C 6.23424200 0.91838200 -0.89882100
H 3.18864500 -6.42186700 4.27915600
H 1.88639900 -5.21834900 4.58077300
H 1.48203200 -6.87434300 4.00703700
C -2.28405600 -5.61472500 0.02643600
H -2.35964900 -5.24750600 -2.09354200
H -1.83851300 -6.32540300 2.02958100
C 5.95723500 1.53868700 0.34257900
C 6.15066900 1.72305100 -2.02616500
C -3.53917700 -4.89776100 0.33456400
C 5.46744900 2.82076500 0.42179800
H 6.01960400 0.96274200 1.26290000

C 5.57798100 3.00690100 -1.97880700
H 6.38502300 1.32006200 -3.01172400
C -4.43804800 -4.49472500 -0.68375900
C -3.71582400 -4.33129900 1.58499200
C 5.17376400 3.56685500 -0.74093400
H 5.16462700 3.18230600 1.40284600
C 5.18656500 3.66200300 -3.18764900
C -5.32039600 -3.45819300 -0.49744800
H -4.38742700 -4.96552200 -1.66438900
C -4.60265200 -3.25943800 1.79838400
H -3.04190500 -4.58814600 2.40227400
C 4.25623200 4.68704800 -0.73955100
C 4.19431500 4.58740100 -3.16948200
H 5.60660500 3.31886800 -4.13307400
C -5.37577100 -2.74618200 0.72570800
H -5.89699300 -3.12297700 -1.35808700
C -4.57406300 -2.55143500 3.03509900
C 3.59903200 5.01545200 -1.93678200
C 3.79897500 5.32010500 0.45697100
H 3.83462900 4.99517100 -4.11088900
C -5.97218000 -1.43860700 0.86364900
C -5.05948400 -1.28655000 3.11893300
H -4.05846700 -3.00181300 3.88320200
C 2.28752300 5.60360500 -1.87979500
C 2.54628000 5.89403700 0.51750900
C 4.69112600 5.43757200 1.64532400
C -5.65613300 -0.65164200 1.98555700
C -6.75625100 -0.80320900 -0.15391200
H -4.91809200 -0.73024600 4.04126600
C 1.70179200 5.95428900 -0.63598100
C 1.47444500 5.60236300 -3.05435700
C 2.10534500 6.39445800 1.86237300
O 5.95455500 5.67888900 1.26703300
O 4.34849400 5.37040800 2.80556700
C -5.73654200 0.78229900 1.89140600
C -6.75642600 0.56938000 -0.28652700
C -7.63771300 -1.60725600 -1.04576400
C 0.27020900 6.18857600 -0.57131100
C 0.12274400 5.67405900 -2.96452600
H 1.92657500 5.41281100 -4.02341400
O 1.50352000 5.41750000 2.54517100
O 2.26166100 7.52256200 2.26213600
C 6.89666500 5.74780200 2.33037700
C -6.03225200 1.39231400 0.64824800
C -5.33317300 1.60619800 2.98582900
C -7.69360600 1.14165900 -1.31781400
O -7.81456100 -1.40907500 -2.22781200
C -0.51619900 5.86396700 -1.70744600
C -0.45841900 6.58750000 0.57798100
H -0.49897300 5.54208200 -3.85011200
C 1.15026800 5.75813600 3.87949400
H 7.85603200 5.98195800 1.86542300

H 6.61438000 6.52367300 3.04952400
H 6.95167000 4.78445600 2.85315800
C -5.51512200 2.72445000 0.40124500
C -4.95786300 2.89423500 2.78962200
H -5.28261500 1.18384500 3.98583500
O -7.40857400 1.71240200 -2.34151300
C -1.89427500 5.60943800 -1.56797600
C -1.80707900 6.34949600 0.68714000
H 0.03758600 7.08784500 1.40259900
H 0.42735300 6.58390000 3.89352700
H 2.04611700 6.05551500 4.43555200
H 0.70862300 4.85823000 4.31177200
C -4.89497100 3.42763700 1.47045200
C -5.36590300 3.28561700 -0.89040700
H -4.59889800 3.50147000 3.62051500
C -2.53438400 5.70636900 -0.34534300
H -2.40751000 5.19194200 -2.43376800
H -2.31263800 6.62898400 1.61080700
C -4.05892700 4.52867800 1.21267100
C -4.53038500 4.35483200 -1.11461500
H -5.83428500 2.80723200 -1.74376700
C -3.77346700 4.94639100 -0.07638600
H -3.50214300 4.93289000 2.05787200
H -4.40193300 4.69844000 -2.13997200
O -8.24502700 -2.58806400 -0.36302800
O -8.95678400 0.90668500 -0.93269200
C -9.94222300 1.22484700 -1.91236400
H -10.90507200 0.99009400 -1.45418400
H -9.89264600 2.28449600 -2.18361000
H -9.77851200 0.61570700 -2.80890900
C -9.05363300 -3.45427600 -1.14909100
H -8.44202700 -3.97277500 -1.89817500
H -9.48944100 -4.17239800 -0.45212100
H -9.83821000 -2.89088500 -1.66554100

***Syn*-Trimer 7**

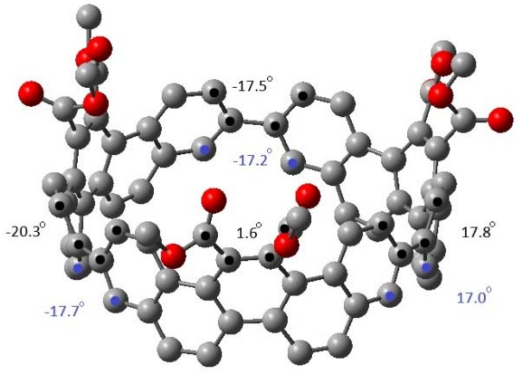

Calculation results at DFT-optimized geometry:

Total Energy: -2448041.285 kcal/mol

SCF Convergence: 0.51E-08

Enthalpy (298 K): -2447331.141 kcal/mol

Entropy (298 K): 379.853 eu

Free Energy (298 K): -2447444.335 kcal/mol

Three lowest frequencies: 11.4, 15.2, 16.8

Predicted change in Energy upon next optimization step: -7.742855E-09

C -5.22730800 -3.43027400 -0.43369400
C -5.56381200 -2.93145100 -1.71386800
C -5.90488000 -2.82027700 0.67883700
C -6.06862200 -1.58843500 -1.83895600
C -5.21056700 -3.70670700 -2.86142400
C -4.17875700 -4.42326600 -0.34109900
C -6.46676400 -1.57005200 0.54400400
C -6.30447000 -0.83579600 -0.67556800
C -6.13281800 -0.91792300 -3.10121900
C -4.35027300 -4.75371900 -2.76618000
H -5.60865400 -3.43168400 -3.83531400
C -3.69510200 -5.03480600 -1.53040800
C -3.42256000 -4.62505500 0.83453900
C -7.28977500 -1.02827200 1.65918400
C -6.19864100 0.60022700 -0.73156700
C -6.17079100 0.43931800 -3.17417700
H -6.08329500 -1.49435900 -4.02173400
H -4.05827800 -5.31231400 -3.65560300
C -2.47524500 -5.73649100 -1.52601200
C -2.20225500 -5.25678900 0.79352700
H -3.74155300 -4.17021900 1.76630500
O -8.33844400 -0.33339400 1.19618400
O -7.07239900 -1.19863200 2.83911100
C -6.06155200 1.23450800 -1.99321700
C -5.96816100 1.38952200 0.41727500
H -6.16265400 0.93823200 -4.14337800
C -1.65676100 -5.76684000 -0.40667300
H -2.12666000 -6.14085500 -2.47714800
H -1.60288000 -5.25693200 1.70127800
C -9.12847900 0.29776400 2.19587400
C -5.62225400 2.57045000 -2.06933400
C -5.48232100 2.66957600 0.31421500
H -6.03024300 0.94692600 1.41025900
C -0.19784200 -6.00668800 -0.49352200
H -9.94220900 0.79539900 1.66519000
H -8.52844200 1.03175700 2.74846000
H -9.52155700 -0.43905400 2.90426600
C -5.22373600 3.27117800 -0.94133600
H -5.47452200 2.99585400 -3.06285500
H -5.18383100 3.18210700 1.22630800
C 0.47266600 -5.86782100 -1.69626700
C 0.61187400 -6.10012000 0.66299900
C -4.28893200 4.41632100 -1.00104100
C 1.85140100 -5.59802800 -1.75620500
H -0.08040500 -5.80369600 -2.63311800
C 1.96194400 -5.83852700 0.63049700
H 0.15519800 -6.32329400 1.62562900
C -3.48828300 4.62620200 -2.10998200
C -3.97802000 5.19008300 0.14412900
C 2.61672400 -5.46013400 -0.56555700
C 2.44967500 -5.29064700 -3.01068200
H 2.48445000 -5.83225800 1.57922100

C -2.27901700 5.34193100 -2.02974200
H -3.69845200 4.11761300 -3.05092700
C -2.79162800 5.87613400 0.25084000
H -4.67183900 5.21768400 0.98346100
C 3.89748900 -4.78039800 -0.63511500
C 3.63008000 -4.62627100 -3.06592400
H 1.90259400 -5.52690700 -3.92336700
C -1.81867000 5.82980600 -0.77824900
C -1.44563400 5.47266500 -3.17613400
H -2.61825500 6.45351400 1.15228600
C 4.30466900 -4.22731500 -1.87274400
C 4.76113700 -4.53375900 0.48548700
H 4.03267200 -4.34268500 -4.03418100
C -0.39903700 6.09684700 -0.62400600
C -0.15503700 5.87161800 -3.05607200
H -1.85413200 5.20419300 -4.15048500
C 5.23710000 -3.13043800 -1.89905000
C 5.74051100 -3.56437300 0.42602300
C 4.72425500 -5.42002800 1.69845300
C 0.43712800 6.06035500 -1.76917600
C 0.24582700 6.23431200 0.64565100
H 0.46409000 5.92069400 -3.94701400
C 5.80414700 -2.67255400 -0.69782700
C 5.43910900 -2.35916300 -3.08740000
O 4.08626700 -4.83237200 2.71755000
O 5.19228700 -6.53025400 1.72812500
C 1.86539300 5.95765200 -1.63606300
C 1.61538600 6.12349200 0.76681500
C 6.21402800 -1.28998200 -0.59676300
C 5.91553000 -1.09086300 -3.02313100
H 5.14224300 -2.75955100 -4.05254600
C 4.14752600 -5.55419400 3.94330300
C 2.42361600 5.77831100 -0.35923300
C 2.73679400 5.85509800 -2.77080200
C 2.23726600 6.43823000 2.08448400
C 6.17168900 -0.48032300 -1.76005200
C 6.41267200 -0.61851800 0.63392500
H 5.99949900 -0.48483100 -3.92514200
H 5.19228900 -5.65579600 4.25695400
H 3.58403100 -4.96326900 4.66779900
H 3.70520200 -6.55152700 3.83023700
C 3.66335700 5.03850700 -0.24567700
C 3.98152600 5.32711600 -2.65645300
H 2.39182300 6.18933600 -3.74616000
O 3.38011700 7.11916900 1.91902900
O 1.77914000 6.16676300 3.17264800
C 6.15578400 0.92148600 -1.64937500
C 6.38121700 0.75334800 0.71674500
H 6.48149000 -1.18028300 1.56366400
C 4.40505700 4.75023000 -1.41881500
C 4.03031900 4.34445200 0.92842200
H 4.62171300 5.22351400 -3.53248000

C 4.08186400 7.41379600 3.12082700
C 6.14695200 1.56340400 -0.42224700
H 5.96621800 1.48150500 -2.56446500
H 6.46922800 1.21369900 1.69936700
C 5.39094000 3.74701700 -1.40892300
C 4.94205400 3.31611700 0.89378800
H 3.48340200 4.49918400 1.85670300
H 4.38544400 6.48574500 3.62167600
H 4.96045600 7.98790500 2.82110500
H 3.45385100 7.99415800 3.80464300
C 5.59039600 2.93226500 -0.30368700
H 5.89027300 3.52717000 -2.35313600
H 5.06869900 2.71767100 1.79317100
C -6.20010200 -3.57248300 1.95101200
O -7.49494000 -3.91874400 1.95967900
O -5.43361600 -3.83989000 2.84290800
C -7.94800700 -4.47481500 3.19166100
H -9.00390200 -4.70935100 3.04300700
H -7.38348900 -5.37938800 3.44068200
H -7.82162200 -3.73915200 3.99432800
C 6.80984900 -3.55905800 1.46243800
O 7.97487600 -3.14319700 0.93962700
O 6.70321800 -3.93061800 2.61053500
C 9.05300400 -3.06608800 1.86300700
H 9.92002700 -2.74256900 1.28407500
H 8.82878900 -2.33787700 2.65277000
H 9.23828400 -4.04067400 2.32665400
C -0.52353000 6.44334200 1.91807700
O -0.84093800 5.26733300 2.46365600
O -0.82378900 7.52091700 2.37160700
C -1.47563700 5.35342400 3.73342500
H -1.63787000 4.32396200 4.05806200
H -0.82519800 5.88410300 4.43725500
H -2.43231400 5.88578800 3.65608000

***TS*-Trimer 7**

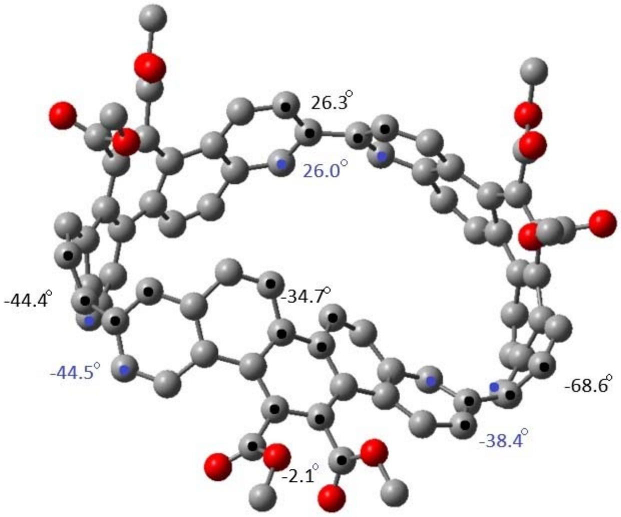

Calculation results at DFT-optimized geometry:

Total Energy: -2448001.459 kcal/mol

SCF Convergence: 0.73E-08

Enthalpy (298 K): -2447292.587 kcal/mol

Entropy (298 K): 370.387 eu

Free Energy (298 K): -2447402.961 kcal/mol

Three lowest frequencies: -12.5, 13.4, 16.8

Predicted change in Energy upon next optimization step: -7.742855E-10

Delta G°: 0.119 kcal/mol

Delta G‡: 41.3 kcal/mol

C 5.54204300 -3.28027100 0.20231600
C 6.03111300 -2.97099200 1.48212400
C 6.05985700 -2.52334700 -0.89632400
C 6.62739700 -1.68146000 1.70815800
C 5.71781400 -3.86653200 2.55835800
C 4.38276600 -4.14421000 0.09897400
C 6.64334200 -1.29020700 -0.68686700
C 6.05046200 -3.07888000 -2.27836000
C 6.82258600 -0.77429300 0.63588900
C 6.72871200 -1.20115000 3.05236000
C 4.74899900 -4.80737200 2.42895100
H 6.24577300 -3.77634400 3.50454500
C 3.94394100 -4.85016800 1.24856400
C 3.50112600 -4.10858000 -1.00487300
C 7.00578300 -0.48136700 -1.89894700
O 6.21387100 -4.41001900 -2.25703000
O 5.95928000 -2.44130100 -3.30460900
C 6.93767700 0.64632100 0.92624300
C 6.63847300 0.12745600 3.30657400
H 6.71551300 -1.90134300 3.88244300
H 4.49307300 -5.45818800 3.26493400
C 2.65330600 -5.40952600 1.29184200
C 2.22156300 -4.60599100 -0.91512200
H 3.76723200 -3.55507400 -1.90407400
O 5.94635000 0.21130000 -2.32377100
O 8.10566700 -0.43815300 -2.39339900
C 6.15965200 -5.04155500 -3.53018500
C 6.58408400 1.06451500 2.23405400
C 7.12970400 1.69503600 -0.01108500
H 6.54005200 0.49567200 4.32764900
C 1.73921300 -5.21283800 0.26831600
H 2.34367600 -5.90251600 2.21468200
H 1.53692000 -4.42667700 -1.74238000
C 6.15625300 0.94069100 -3.52596500
H 6.33718500 -6.10260400 -3.34564900
H 5.17276500 -4.89381200 -3.98684700
H 6.92239100 -4.63131500 -4.20012800
C 5.96152500 2.31242400 2.40344100
C 6.58745700 2.94399200 0.20556500
H 7.63884400 1.50827500 -0.95167500
C 0.27982300 -5.35668200 0.47249200
H 6.45066000 0.25569500 -4.32874500
H 5.20295800 1.41988600 -3.75662200
H 6.94108200 1.69631900 -3.39267900
C 5.80970700 3.21624200 1.36557000
H 5.43713500 2.48732100 3.34599300
H 6.60577700 3.67176500 -0.60730400
C -0.62668000 -5.43330900 -0.61024400
C -0.26626800 -5.16111700 1.73079900
C 4.47227600 3.82024200 1.11325600
C -1.97389800 -5.20307800 -0.44484700
H -0.24985400 -5.62790800 -1.61368000

C -1.63578500 -4.90488700 1.92224100
H 0.38703800 -5.04997000 2.59649000
C 4.13202300 5.09859600 0.62877500
C 3.43697000 2.91962500 1.29573000
C -2.52456600 -4.87478600 0.81700700
H -2.58959500 -5.17486500 -1.34162000
C -2.10866000 -4.49250100 3.20377800
C 2.81919700 5.41959500 0.31540200
H 4.90870000 5.85227100 0.49964100
C 2.16003100 3.15429200 0.77261900
H 3.66606700 1.90242900 1.61108100
C -3.84827600 -4.30673300 0.99737600
C -3.29223200 -3.84266800 3.32494500
H -1.45879000 -4.62378200 4.06882800
C 1.78603200 4.45289000 0.33296600
H 2.61448900 6.41910400 -0.05572800
C 1.34395200 2.01821000 0.50406900
C -4.13844900 -3.61806900 2.19080900
C -4.81418100 -4.22307800 -0.05248600
H -3.58573600 -3.46772100 4.30132400
C 0.41109400 4.61545600 -0.13844900
C 0.24468200 2.15019000 -0.27189500
H 1.67903000 1.04072800 0.84871500
C -5.16123300 -2.60555800 2.20366100
C -5.77751200 -3.23464800 -0.05668200
C -4.86098900 -5.24363500 -1.13901000
C -0.26330800 3.45312300 -0.55859200
C -0.37698400 5.80563300 -0.13070500
H -0.34578800 1.27974700 -0.54727700
C -5.92376400 -2.32823400 1.04001100
C -5.24976200 -1.71185000 3.31616300
C -6.61479800 -3.12508200 -1.29801700
O -4.69856000 -6.47438800 -0.63401700
O -5.05949700 -5.02740700 -2.31432900
C -1.58010800 3.52362200 -1.09969000
C -1.76592600 5.75653200 -0.25961800
C 0.23257700 7.13658600 0.18366300
C -6.65366700 -1.07398000 0.95491600
C -5.80407500 -0.48211900 3.17678500
H -4.76096300 -1.95992600 4.25317400
O -6.02833800 -2.30608800 -2.17663300
O -7.66920900 -3.68485300 -1.47474600
C -4.67803300 -7.52151300 -1.59692200
C -2.43880300 4.56435500 -0.69058100
C -2.02793900 2.49844800 -1.98483400
C -2.49522900 7.04077700 -0.01791400
O 0.10707600 7.73202900 1.22630500
C -6.40686700 -0.09151200 1.94819600
C -7.45747200 -0.64655400 -0.13463700
H -5.74598500 0.24742000 3.98445400
C -6.69522700 -2.20292000 -3.42823600
H -4.58393700 -8.44864900 -1.02871400

H -5.59830700 -7.52116700 -2.18997600
H -3.82151200 -7.40039900 -2.27231000
C -3.86690700 4.23821700 -0.71125200
C -3.34136700 2.38330200 -2.27812800
H -1.28817700 1.84640200 -2.44539900
O -3.36729300 7.23456100 0.79468500
C -6.56613200 1.27408100 1.63941600
C -7.62308600 0.69114500 -0.40980400
H -7.92140800 -1.36949600 -0.79799600
H -7.70097000 -1.78203200 -3.30112600
H -6.77665500 -3.19249800 -3.89112800
H -6.08139500 -1.54005300 -4.04160400
C -4.27921400 3.12542600 -1.49722200
C -4.82378300 4.73619900 0.20781100
H -3.70785900 1.62491900 -2.96886000
C -7.01220500 1.68486700 0.39654200
H -6.12538100 2.00024300 2.32488700
H -8.13698900 0.98708600 -1.32516500
C -5.51544500 2.50825100 -1.27446300
C -5.99390100 4.04624600 0.47780600
H -4.57141900 5.59298200 0.82367500
C -6.32523000 2.85790700 -0.20597700
H -5.70305900 1.56976800 -1.79760800
H -6.61767900 4.37905700 1.30809200
O 0.92597100 7.60661300 -0.86611900
O -2.01672300 8.01088300 -0.81637400
C -2.48488000 9.32069500 -0.50642900
H -2.04548800 9.98018300 -1.25810500
H -3.57787600 9.36312800 -0.54915000
H -2.15375400 9.60388100 0.50037300
C 1.47904300 8.90352600 -0.67337600
H 2.18312300 8.90559800 0.16798300
H 1.99080600 9.15454700 -1.60440000
H 0.68308900 9.62770200 -0.46346500
